# Supplementary figures and images for: Development of label-free cell tracking for discrimination of the heterogeneous mesenchymal migration
Source: PLoS One. 2025 Mar 31;20(3):e0320287. doi: 10.1371/journal.pone.0320287 (PMC11957292; doi:10.1371/journal.pone.0320287)

S1 Fig

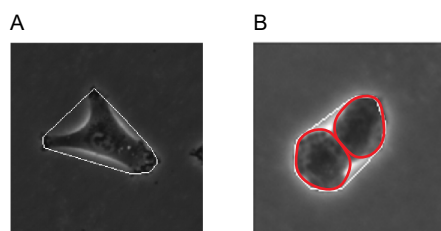

Supplement: S1 Fig — The process did not work when a cell exhibited a constricted shape due to failure to trace the cell outline during the smoothing process (A). Another case is when cell-cell contacts occurred during the cell tracking. The cells (red outlines) were recognized as a single cell as indicated with the white outline (B). (PDF) [file pone.0320287.s001.pdf]

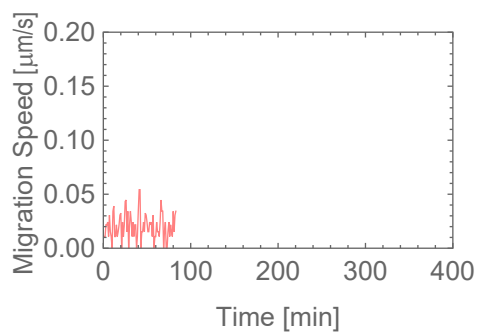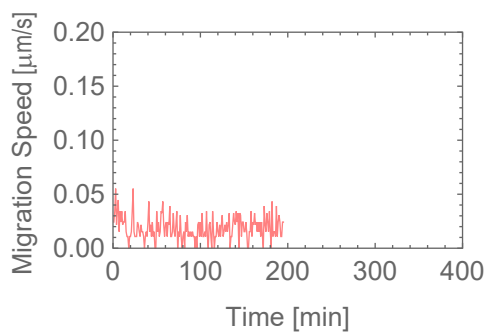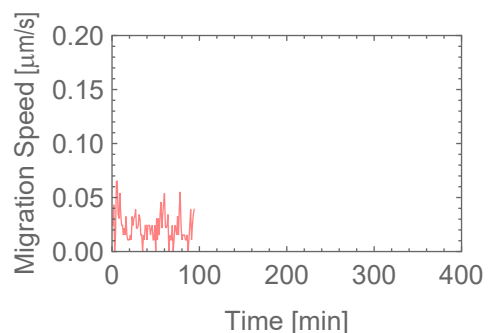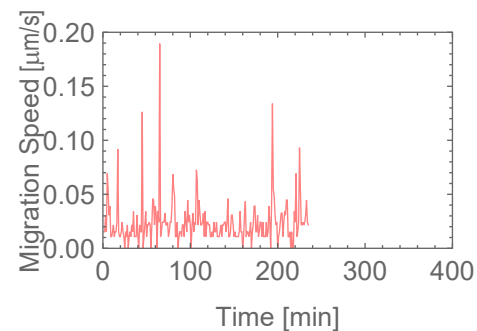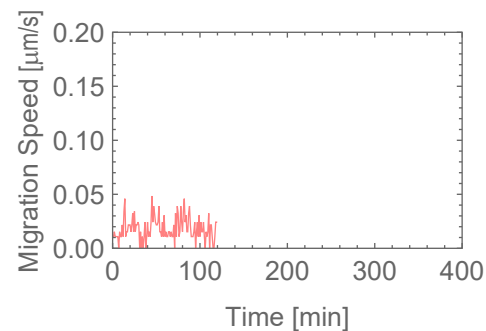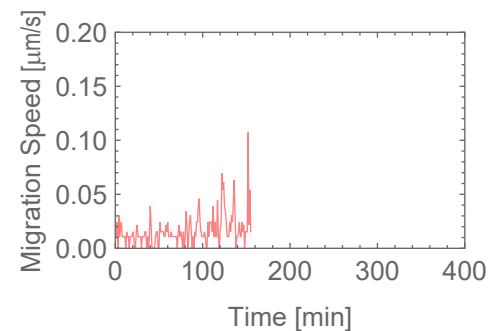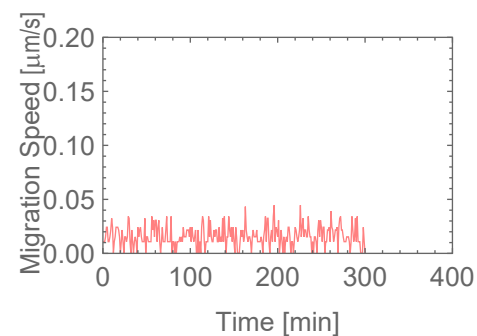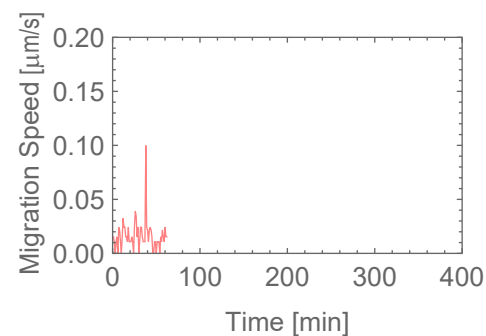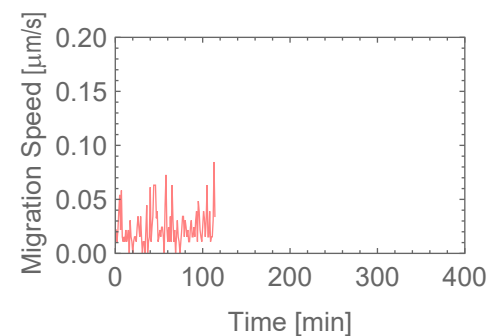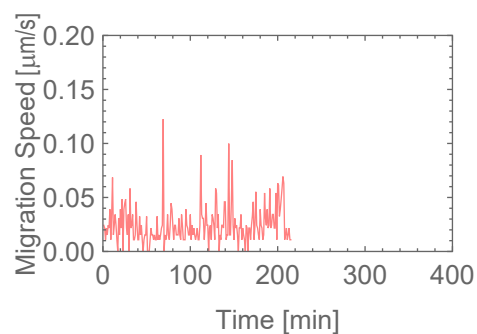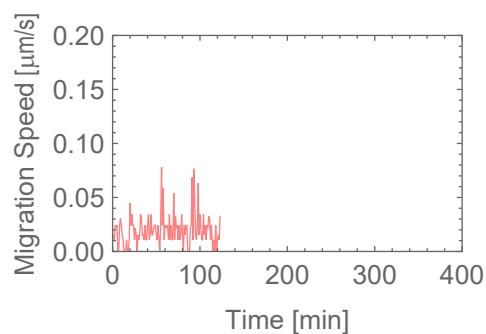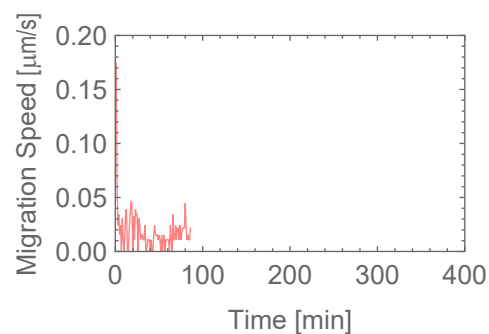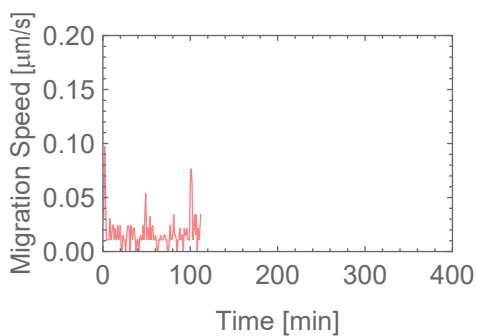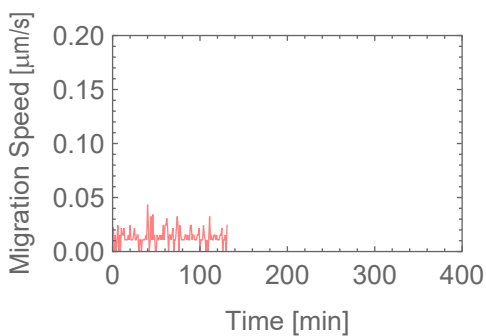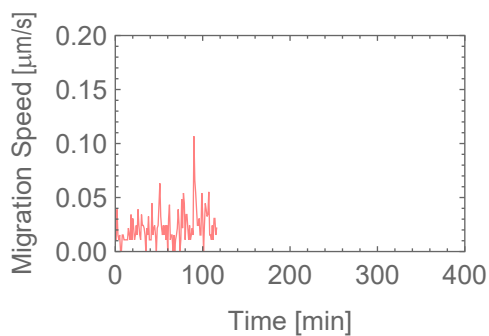

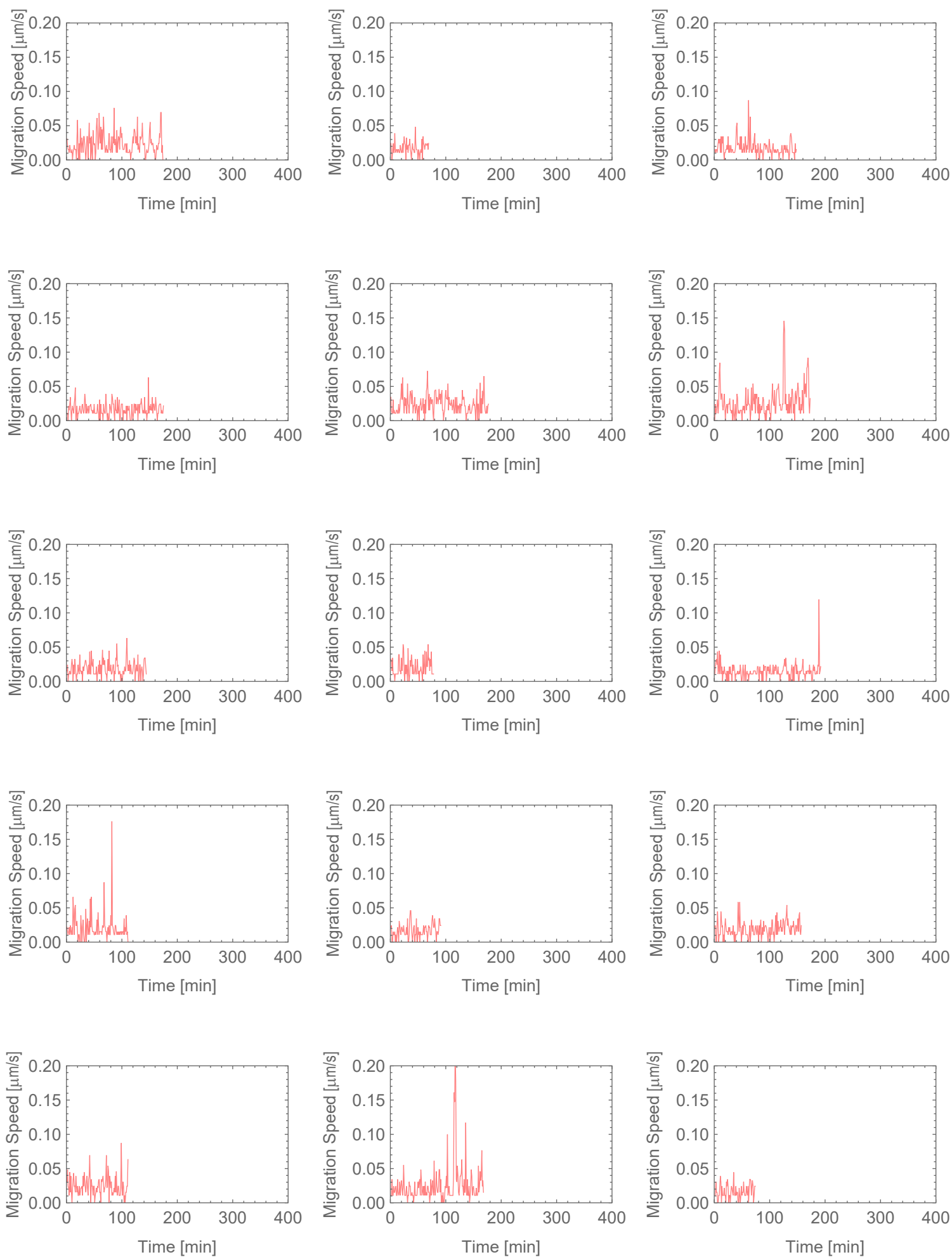

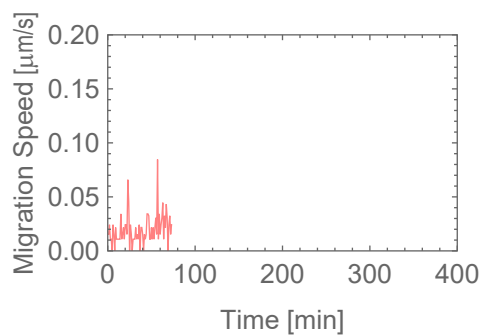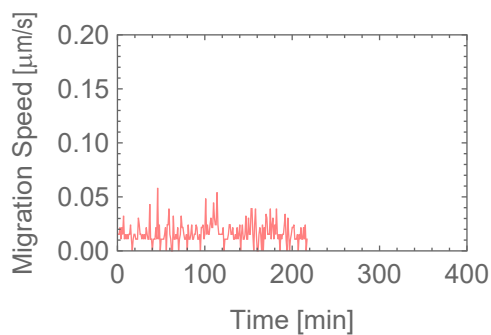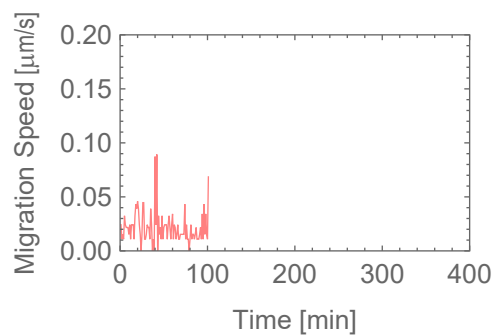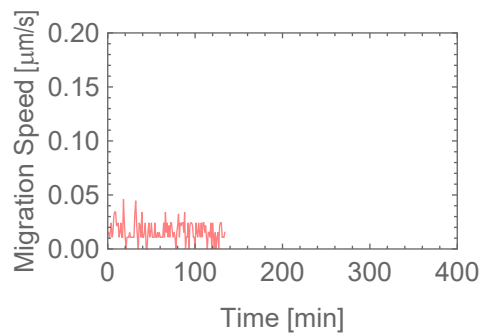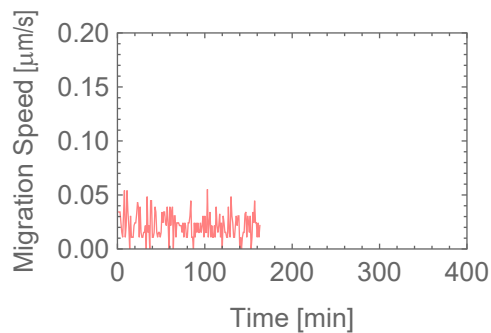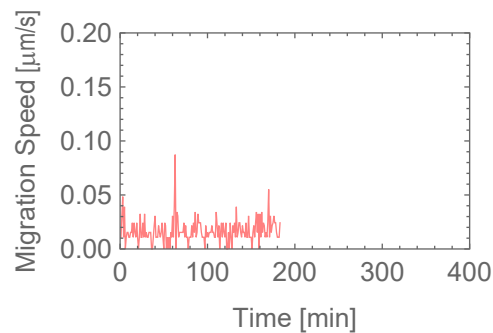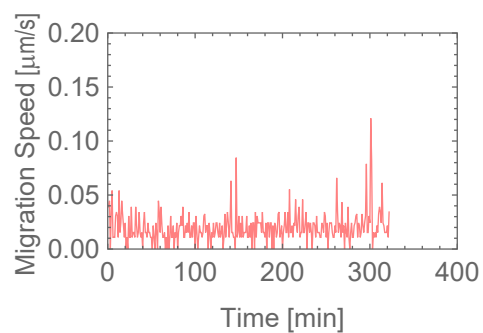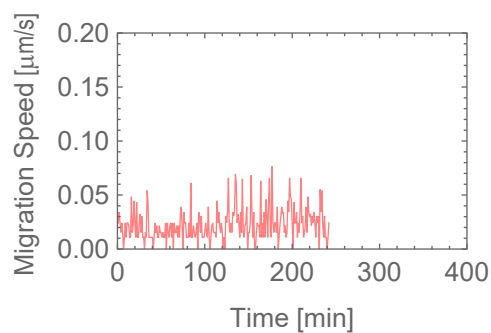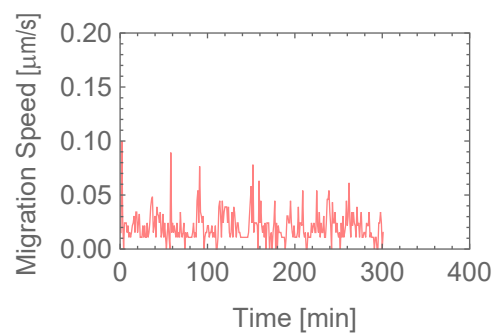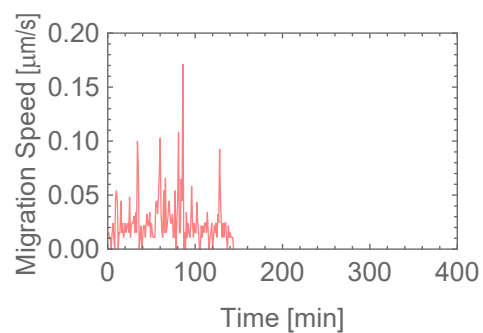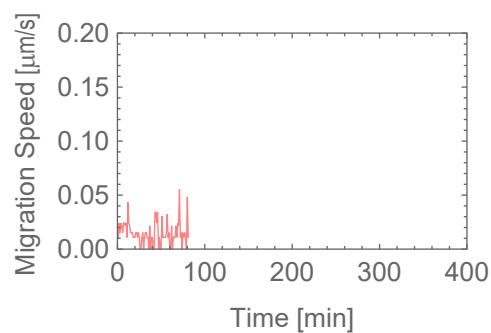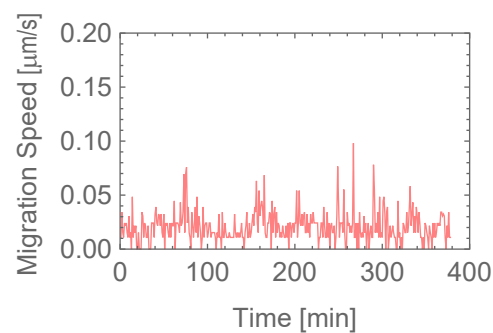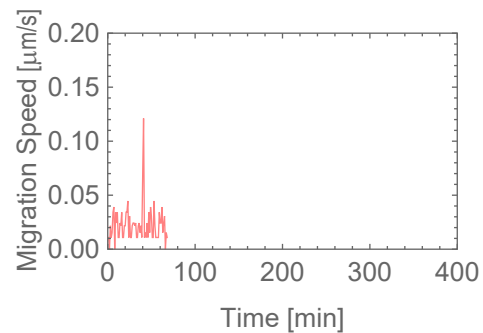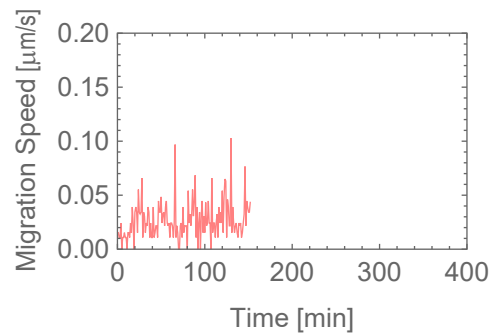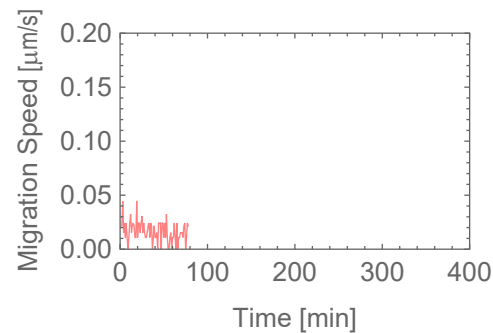

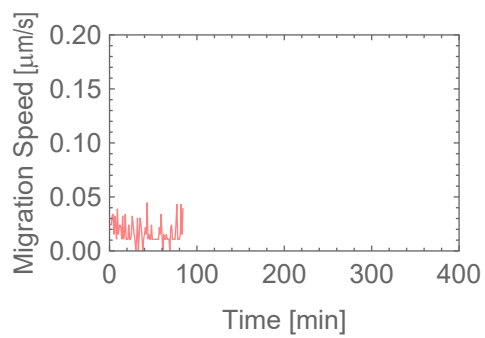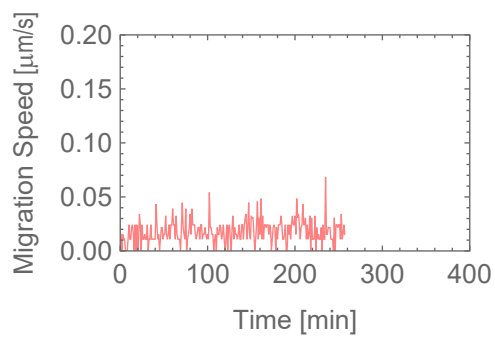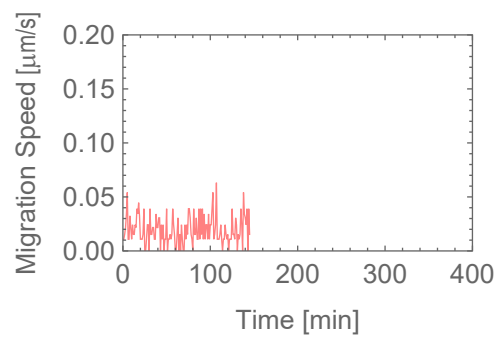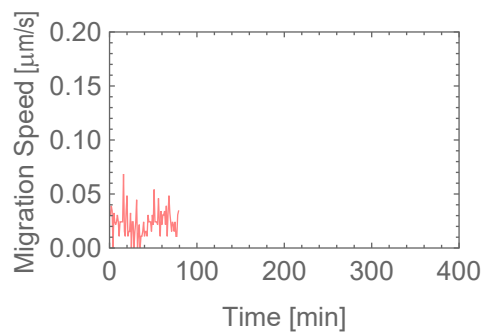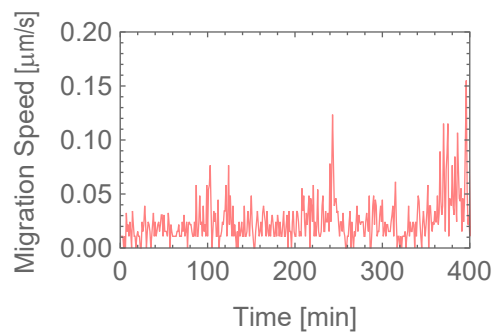

Supplement: S2 Fig — The data were obtained from the same trajectories used in the discriminant analysis. (PDF) [file pone.0320287.s002.pdf]

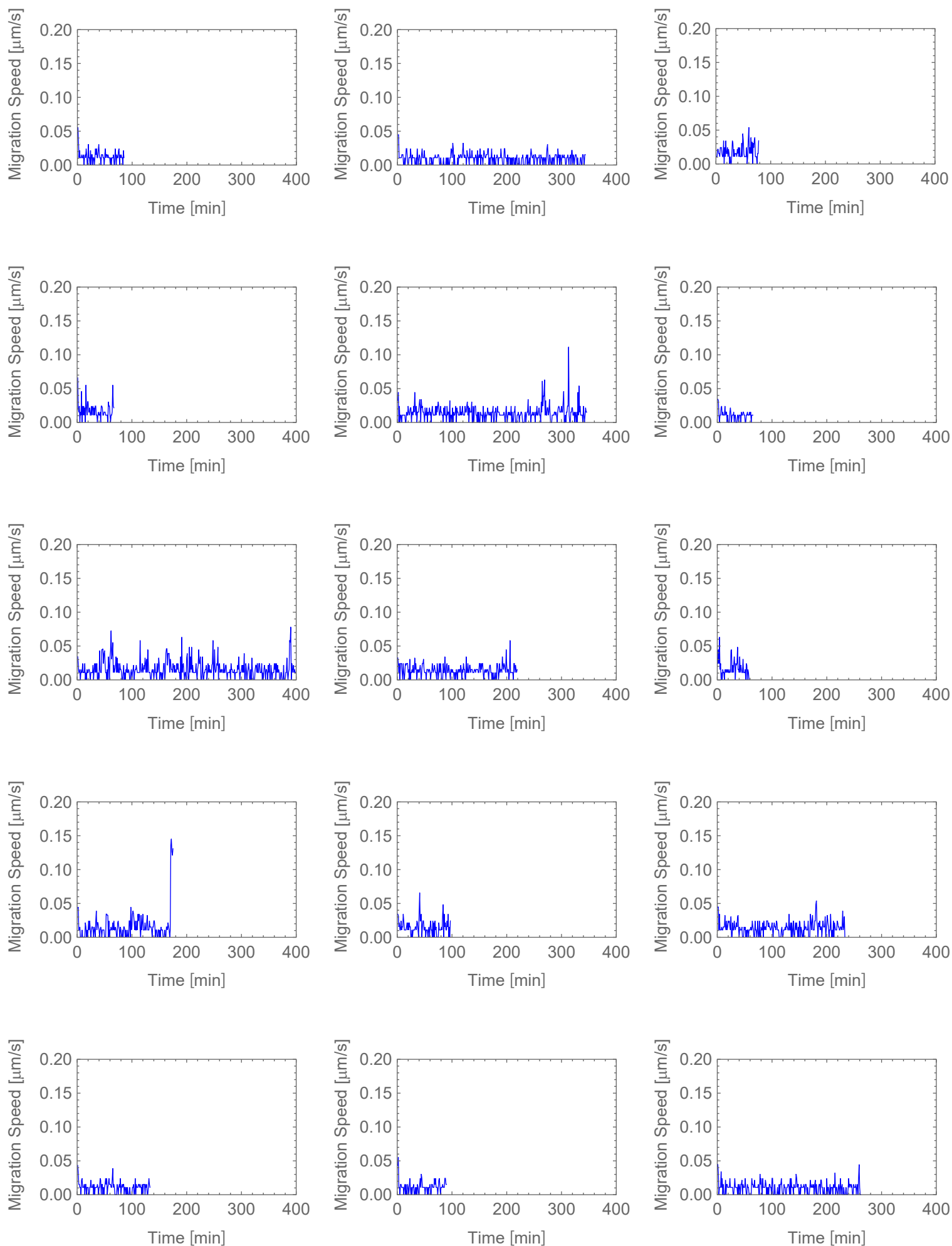

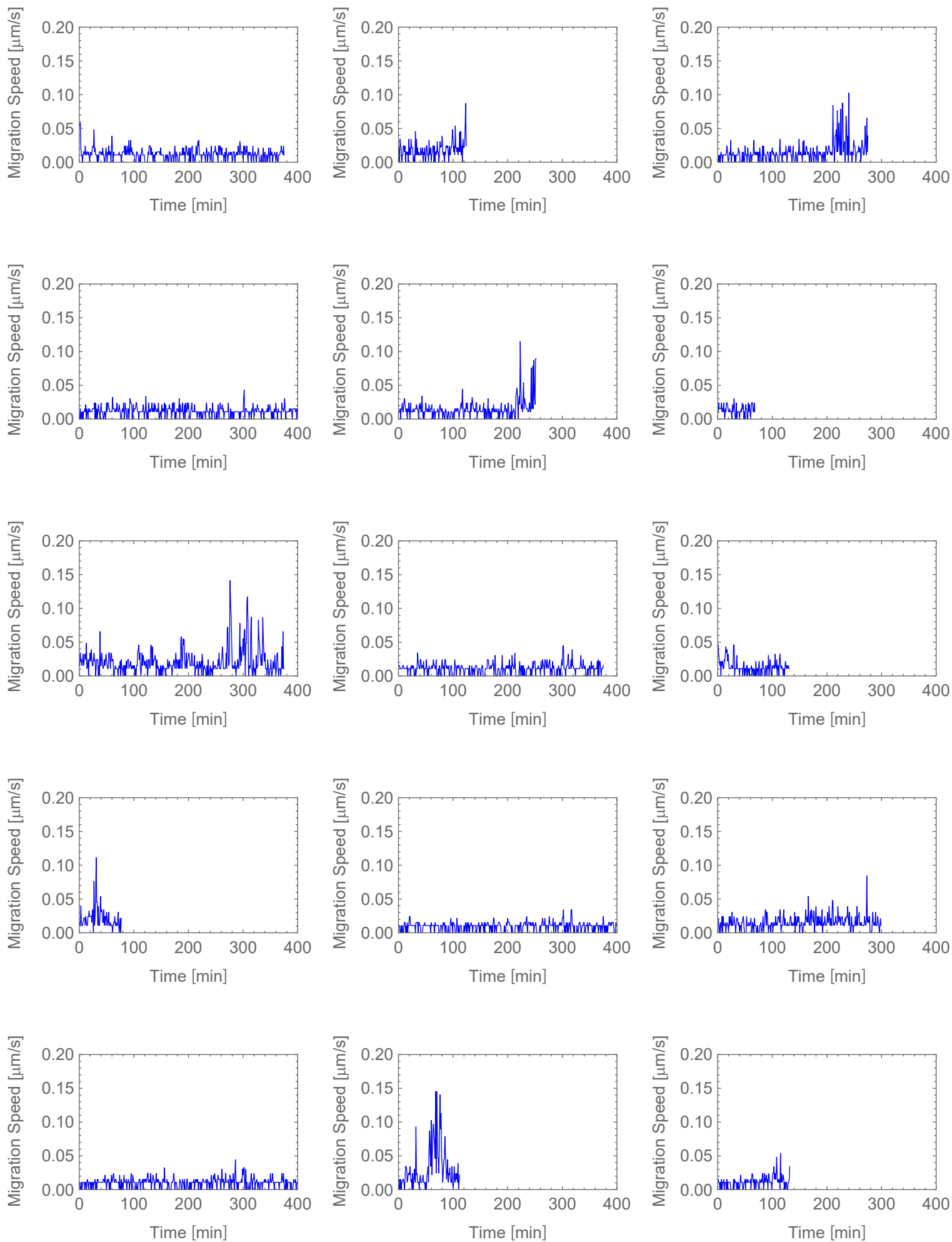

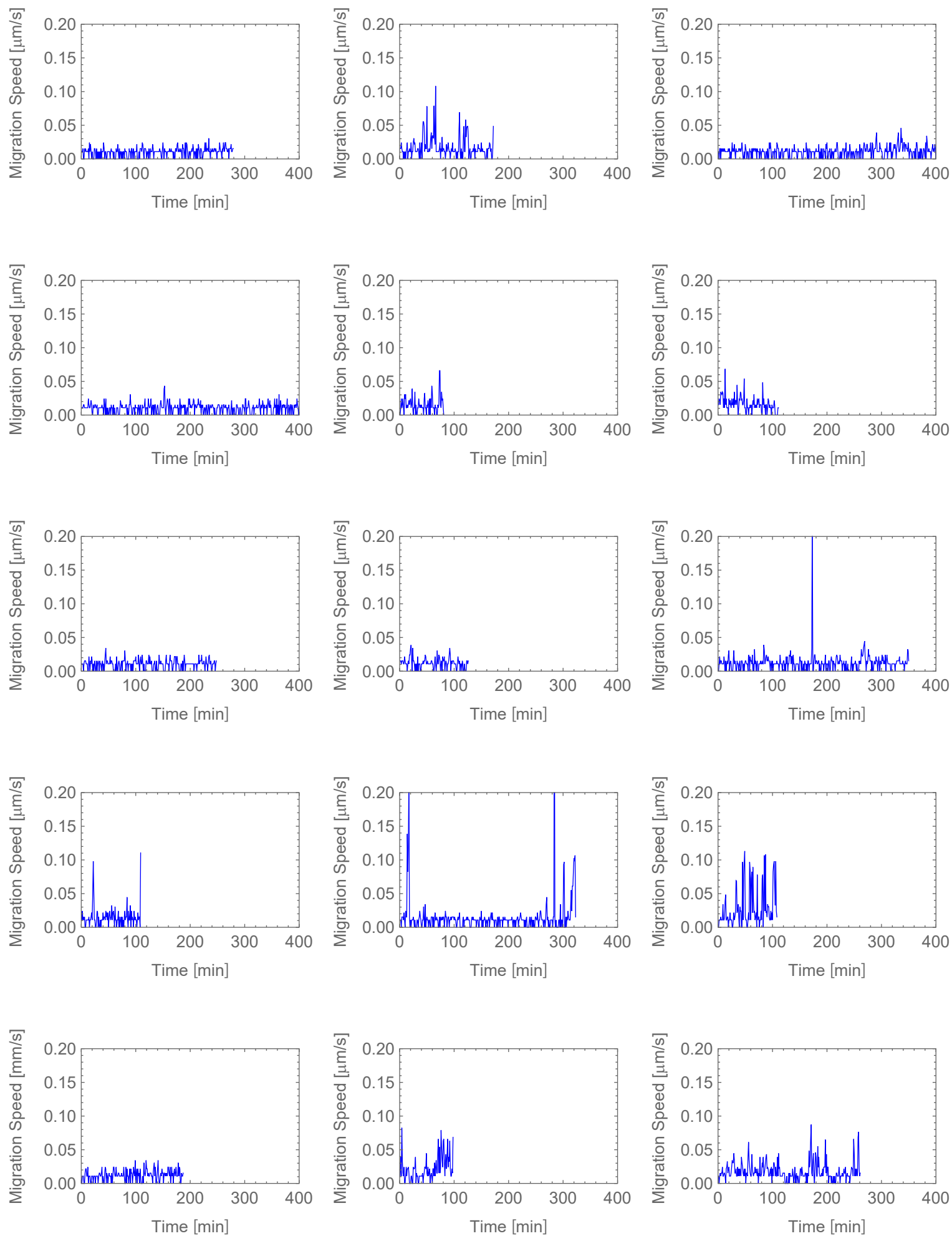

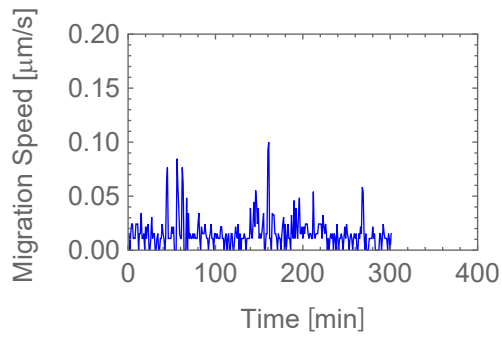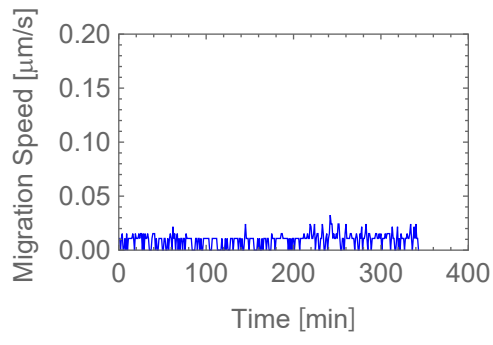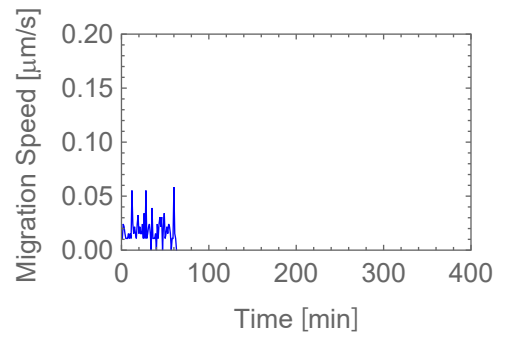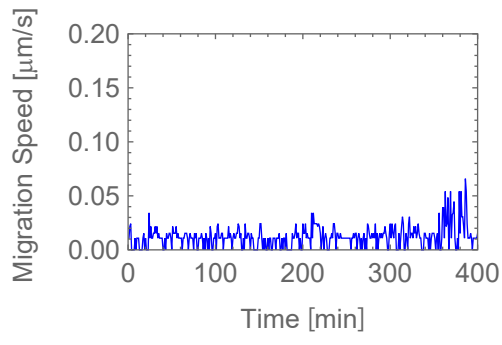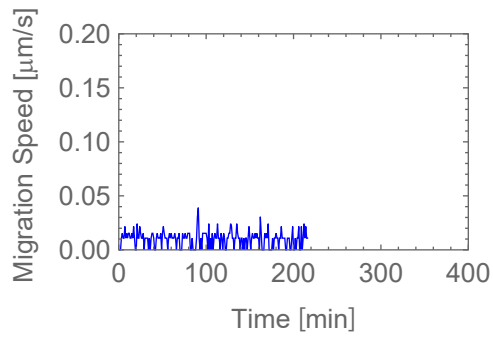

Supplement: S3 Fig — The data were obtained from the same trajectories used in the discriminant analysis. (PDF) [file pone.0320287.s003.pdf]

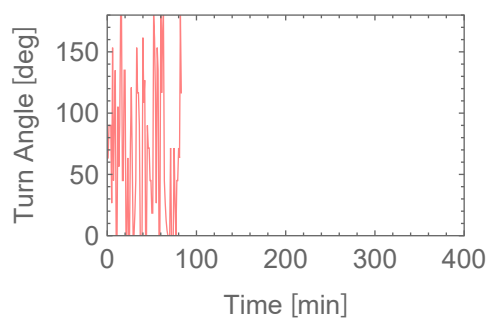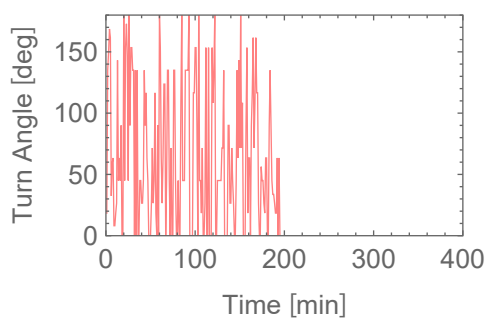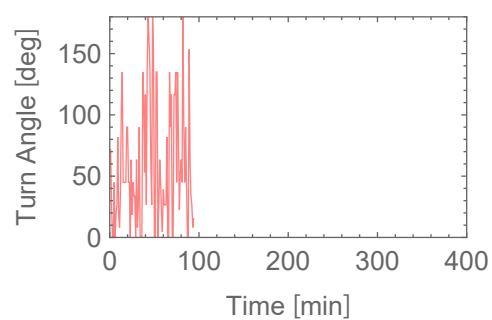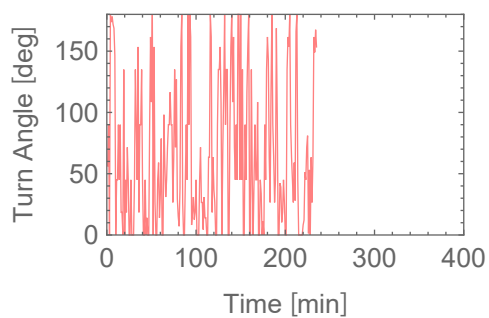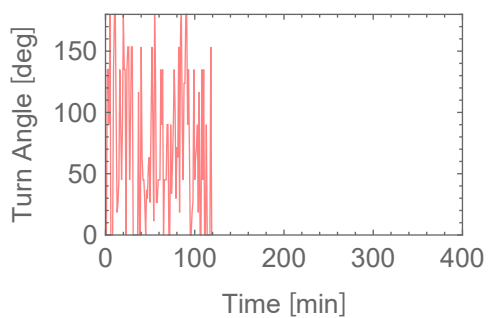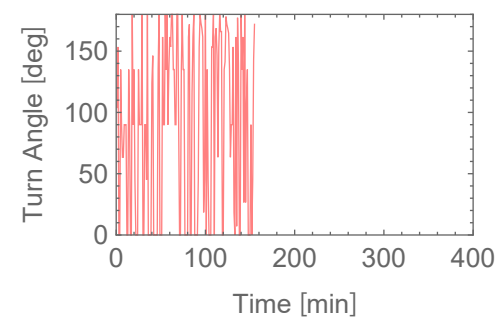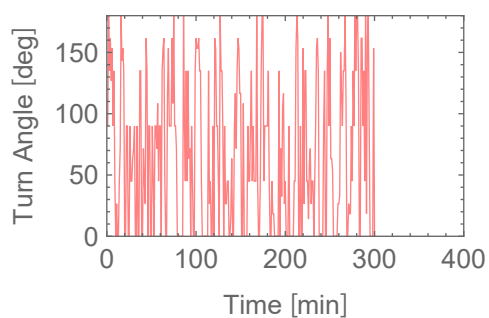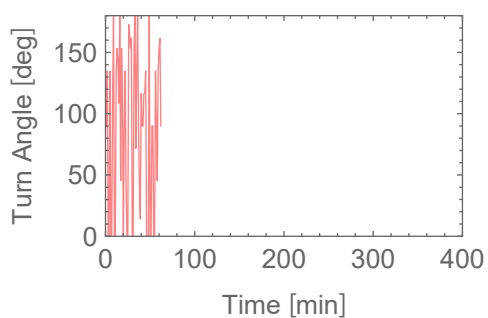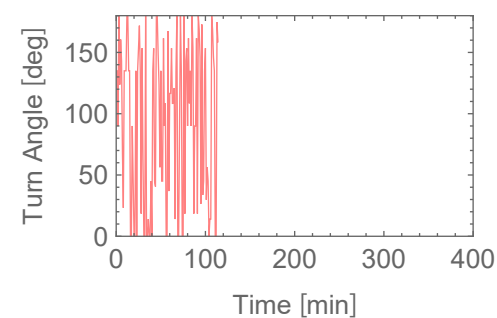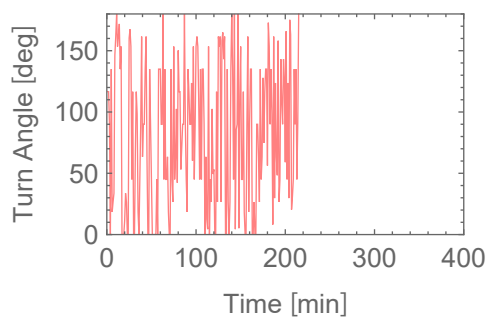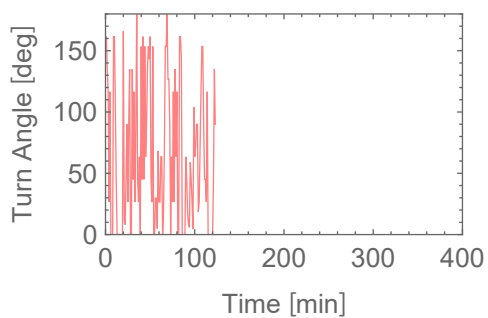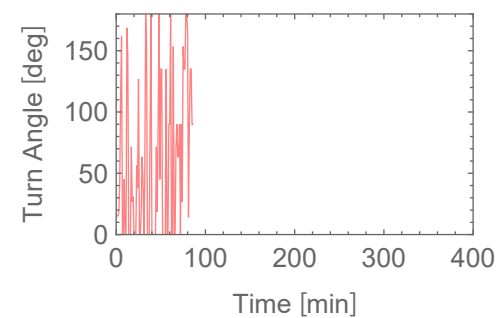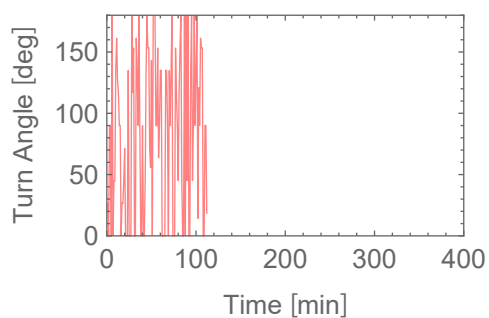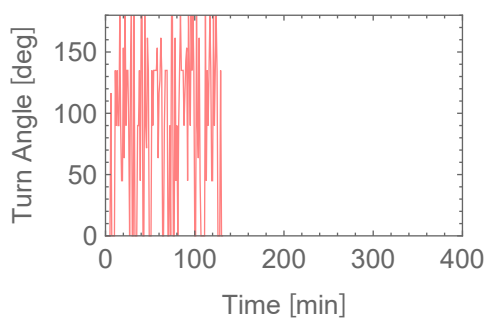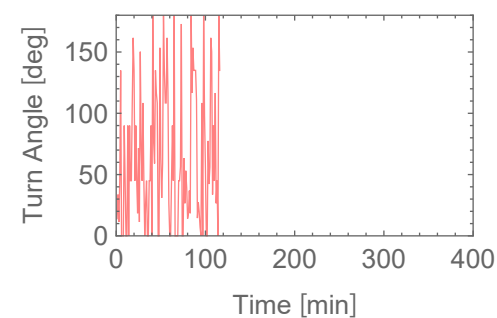

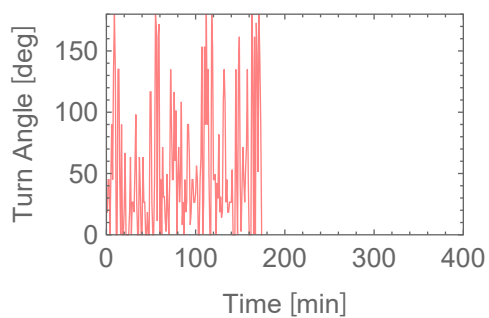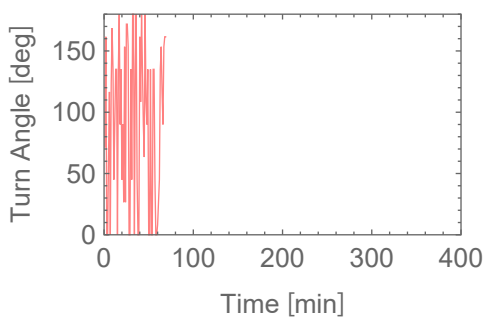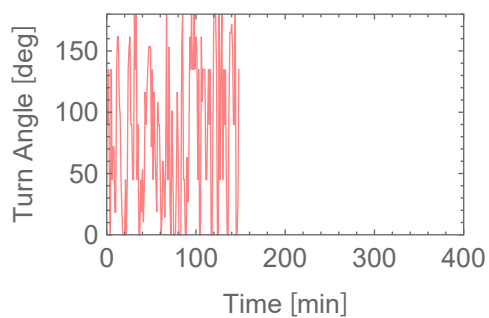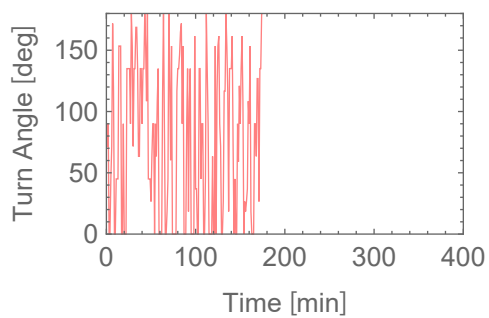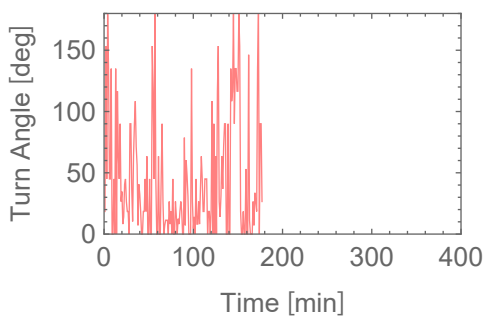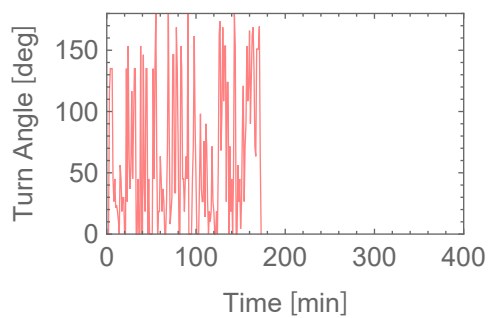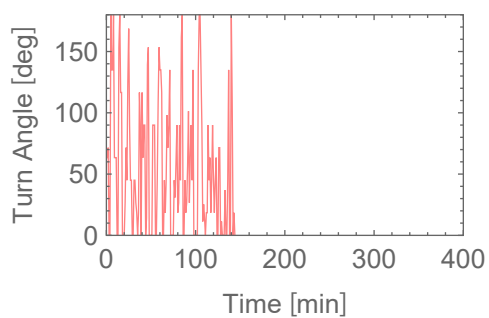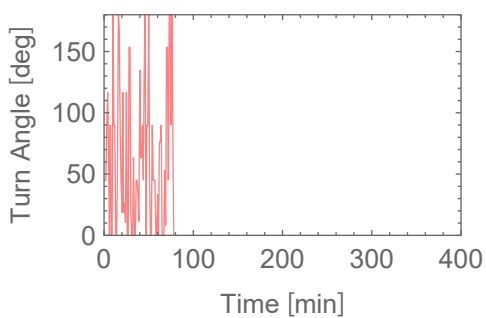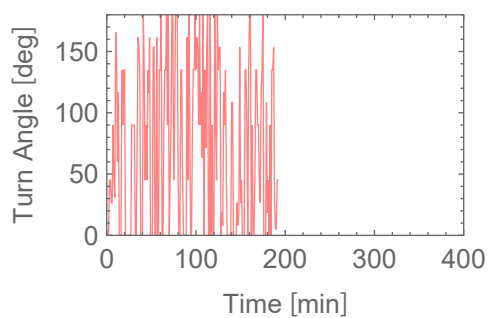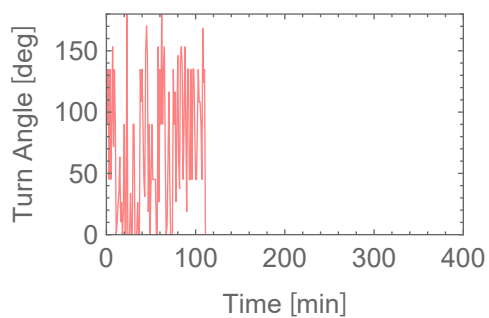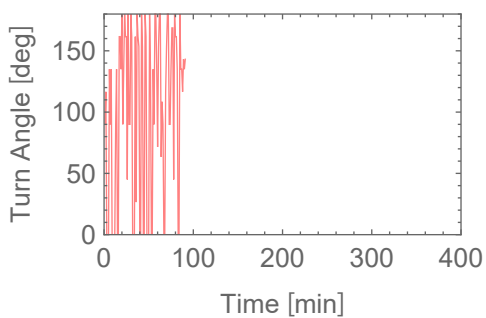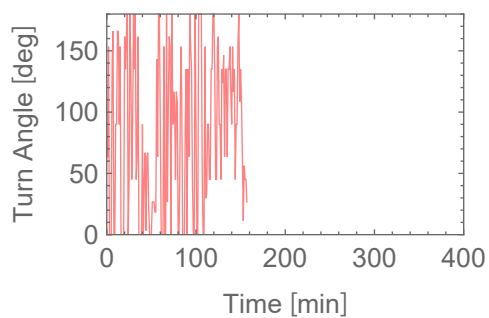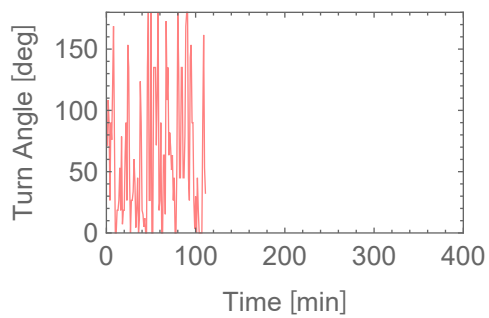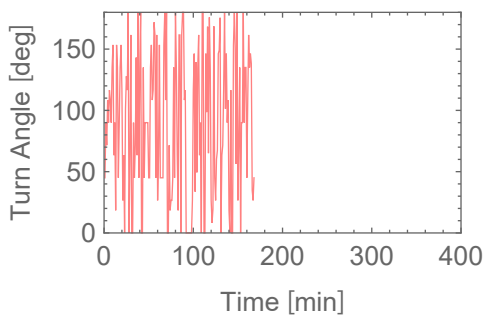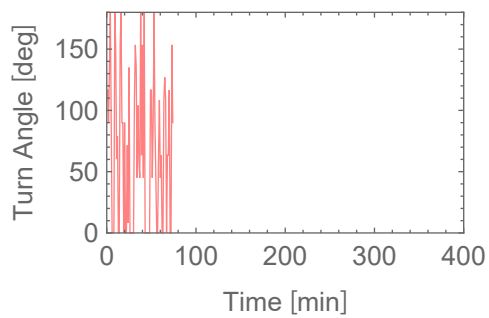

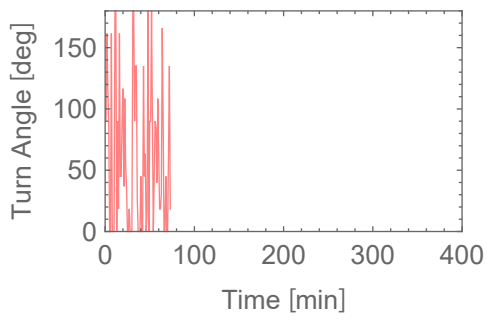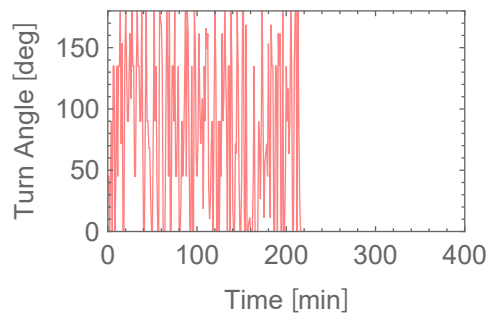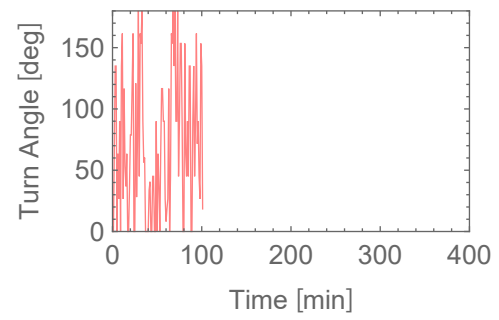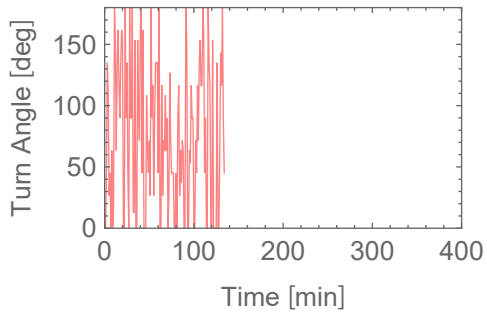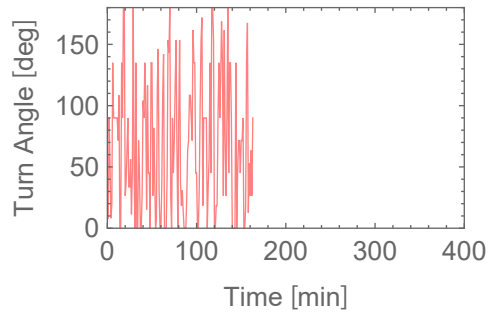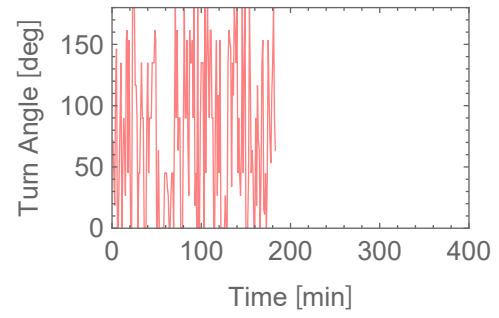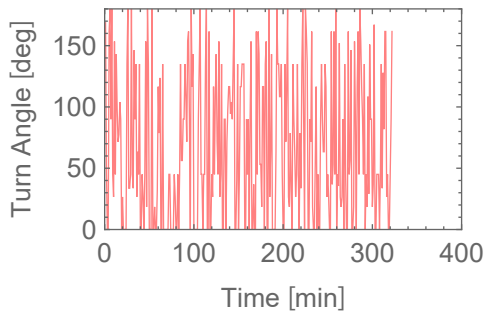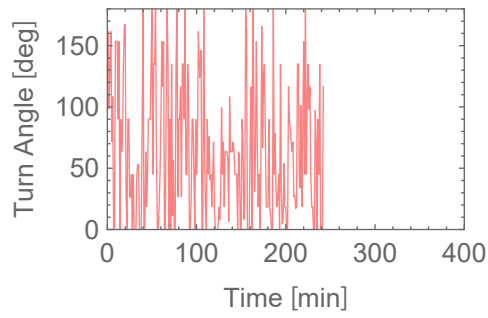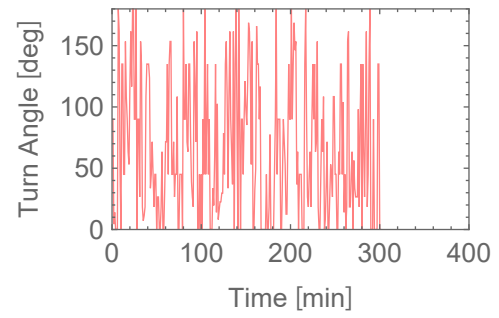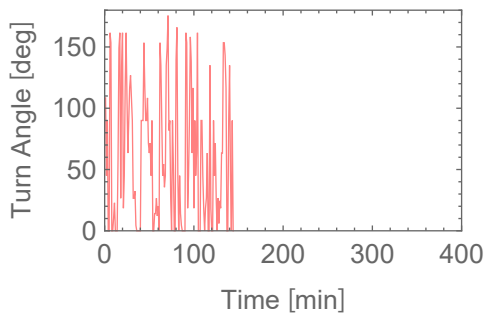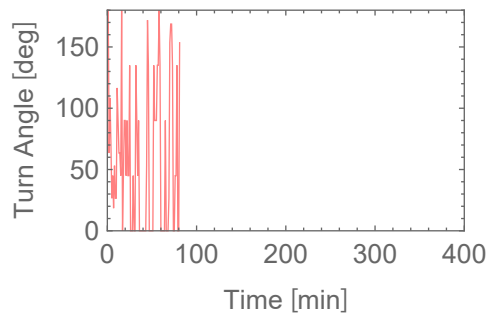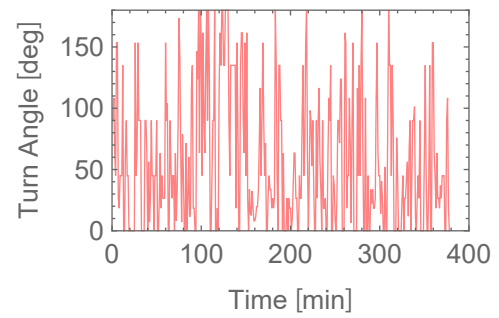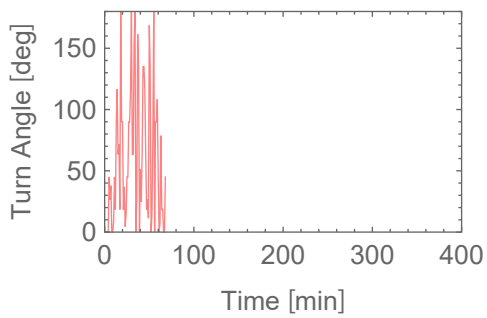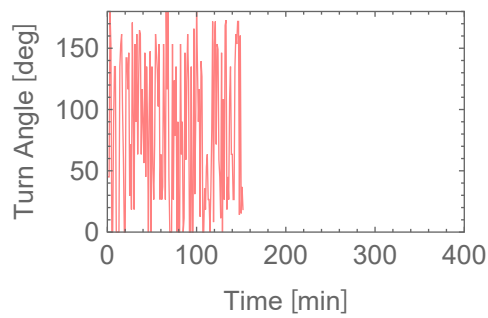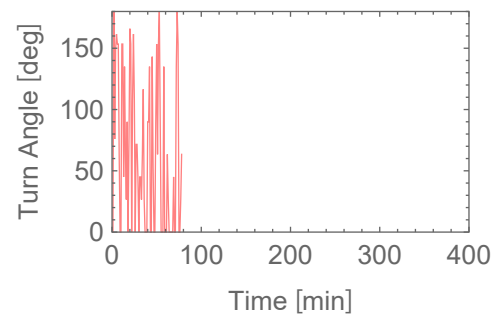

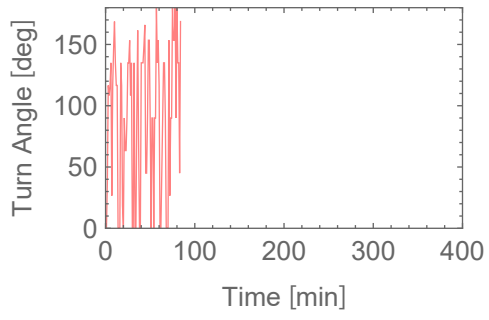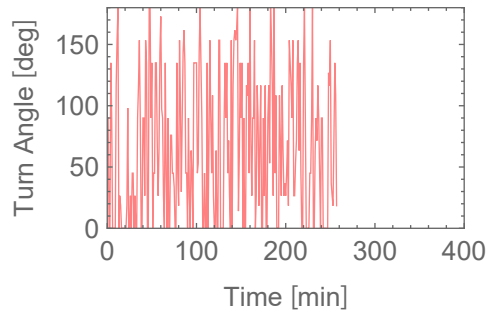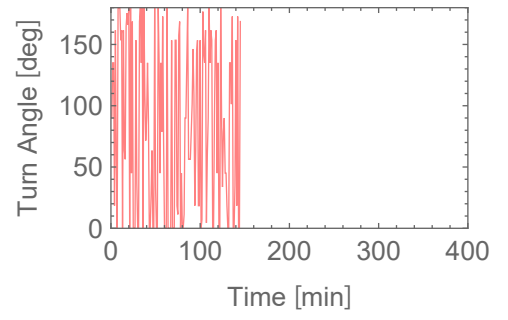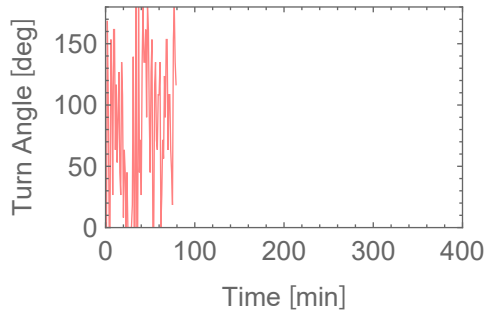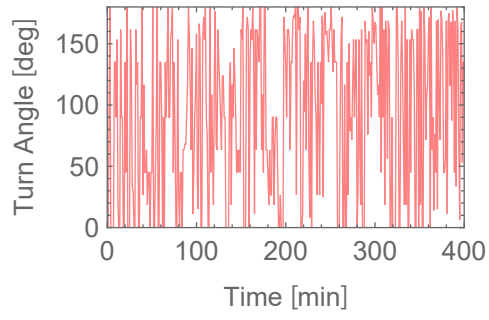

Supplement: S4 Fig — The data were obtained from the same trajectories used in the discriminant analysis. (PDF) [file pone.0320287.s004.pdf]

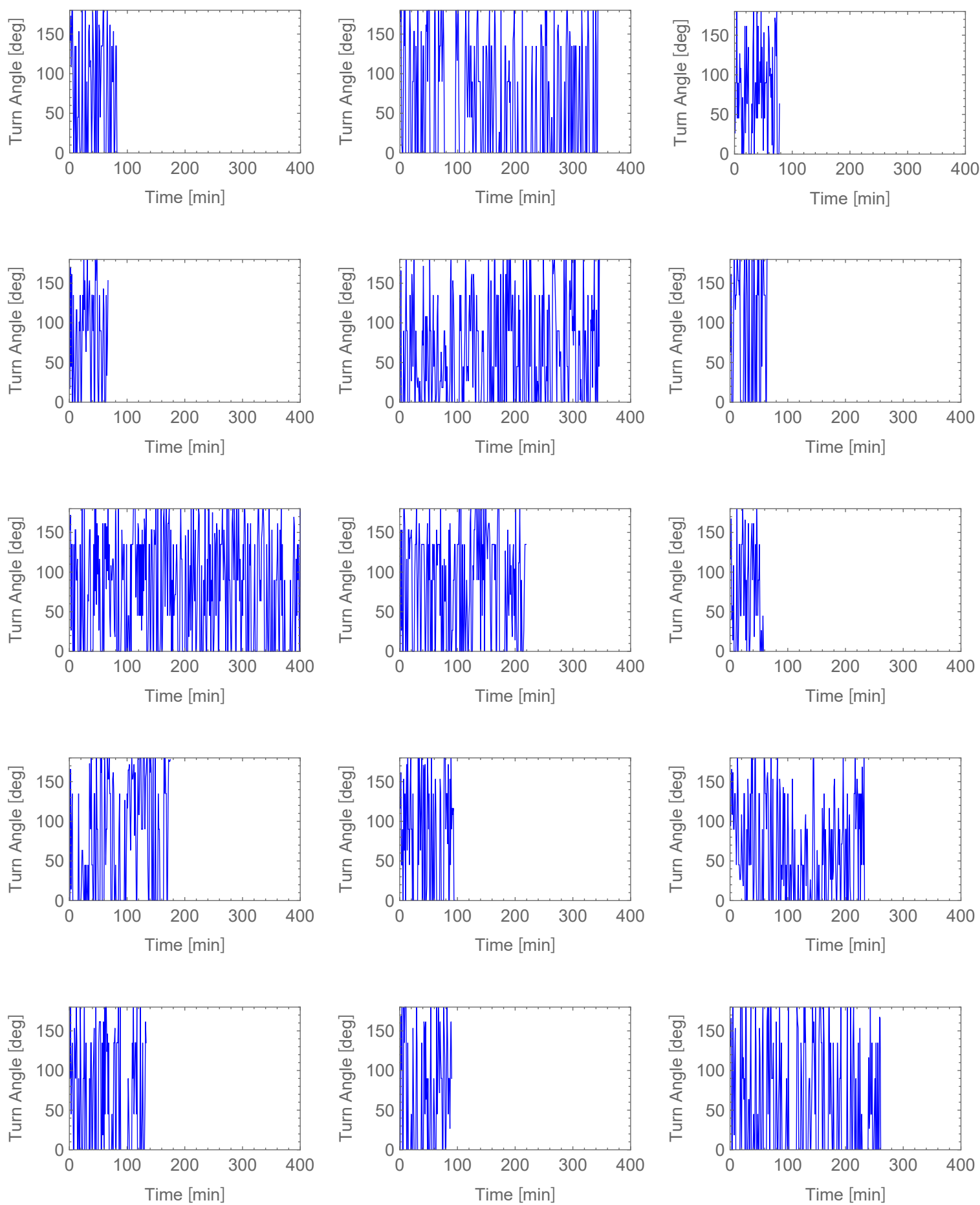

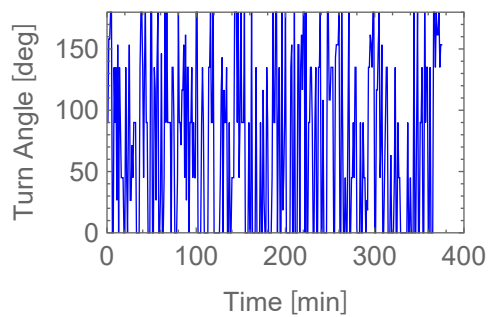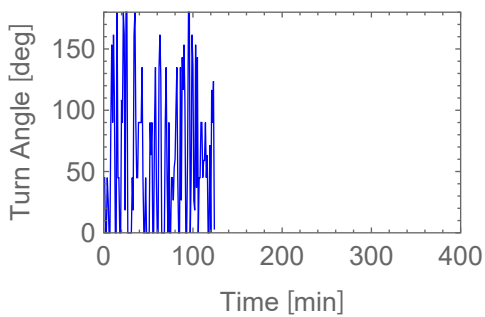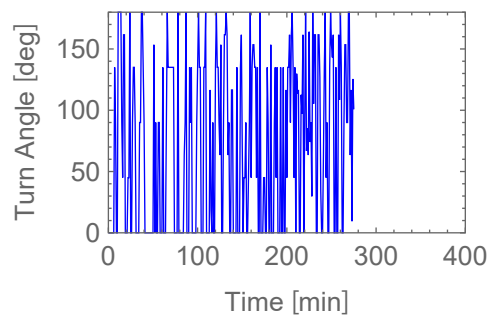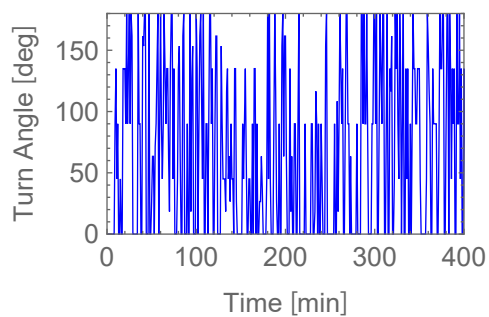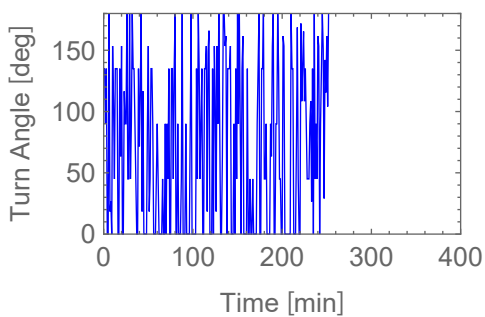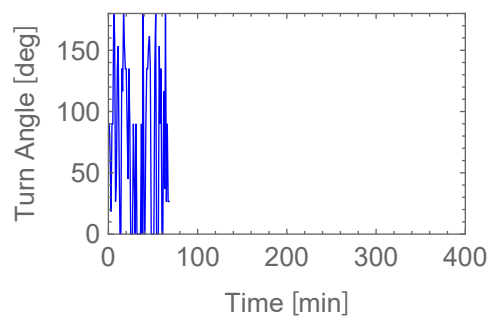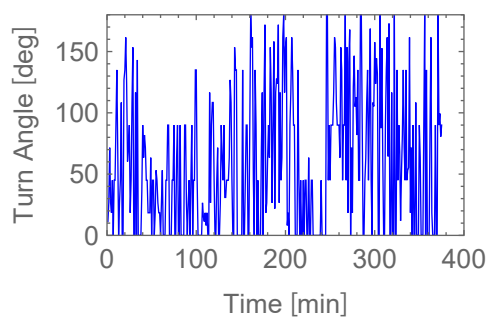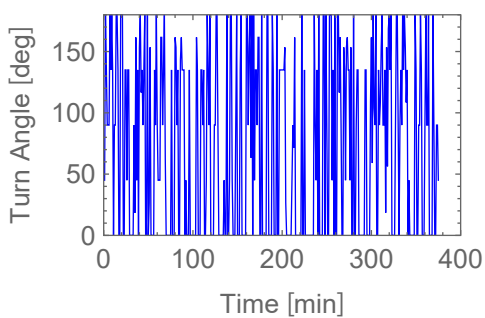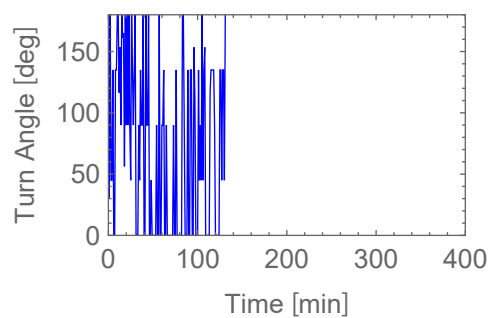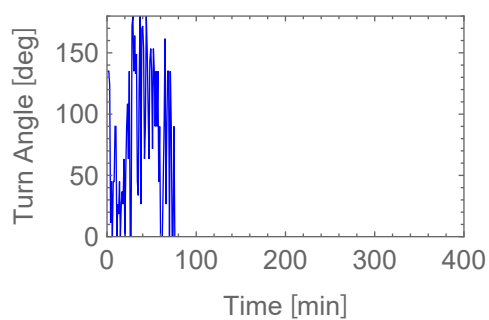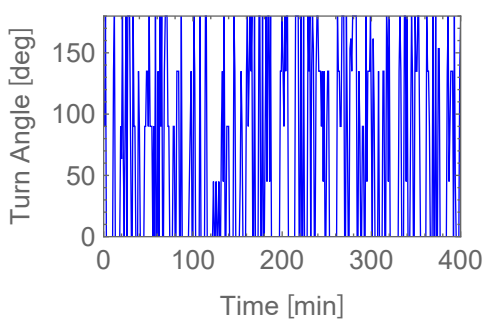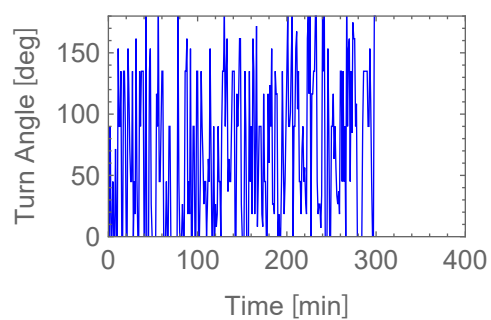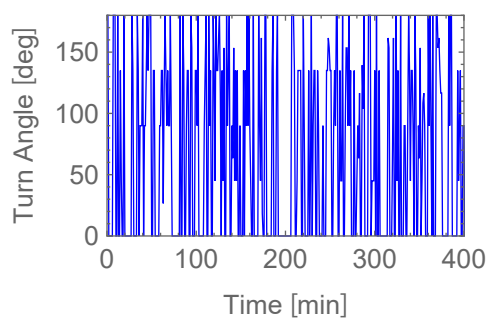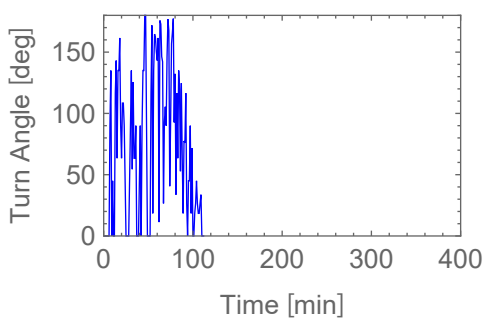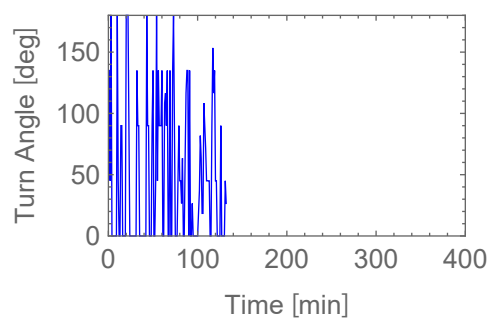

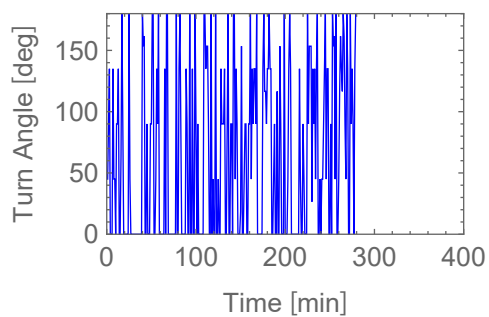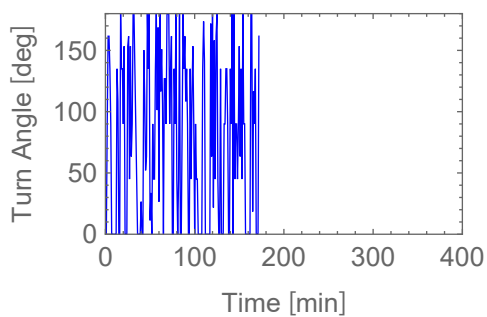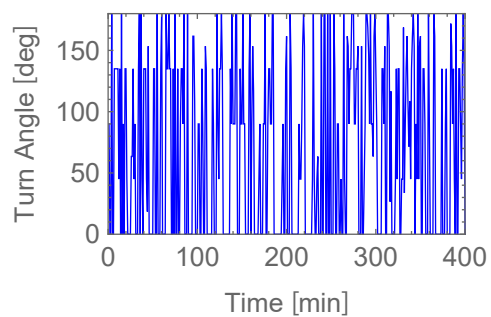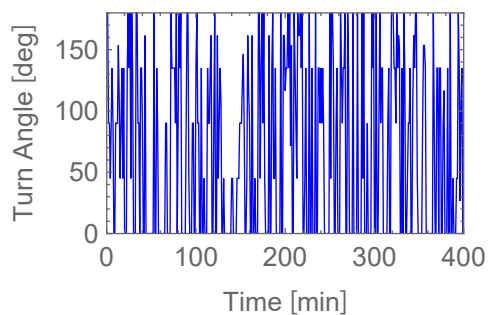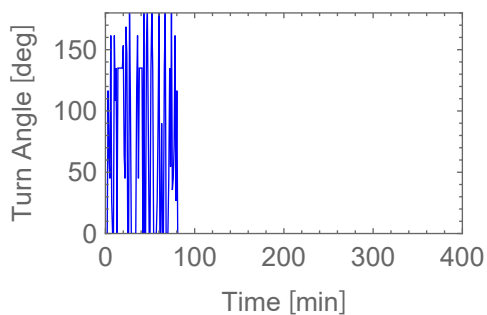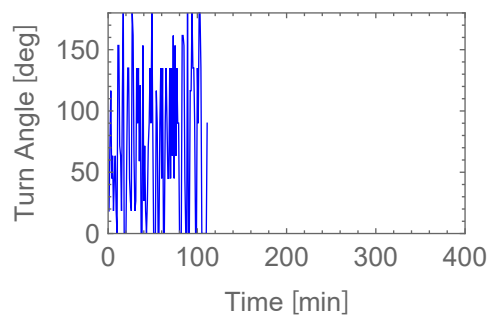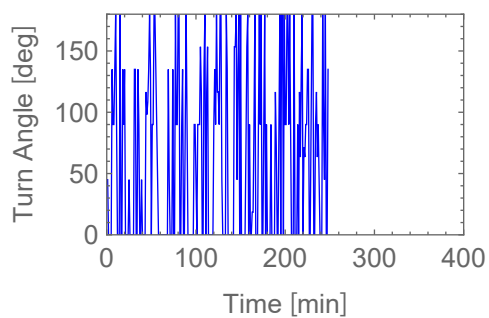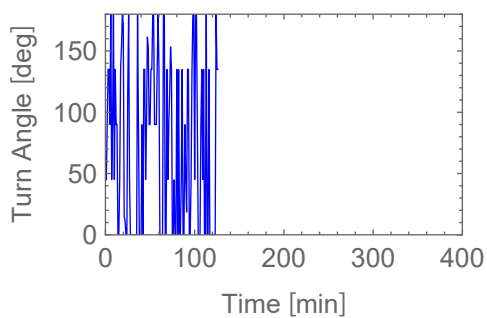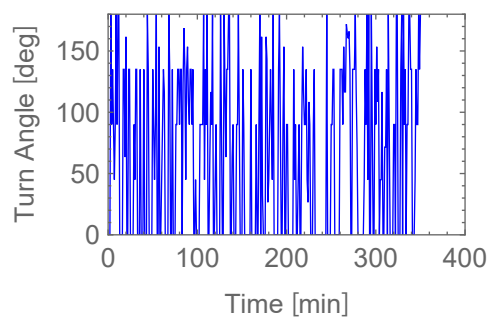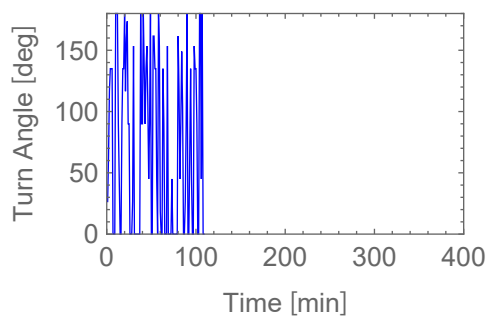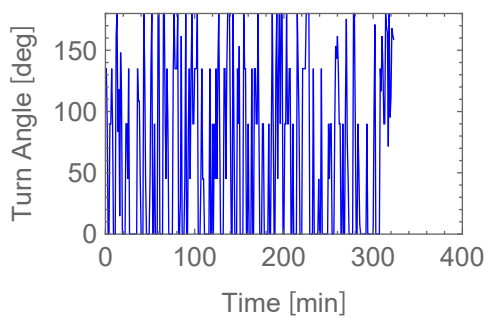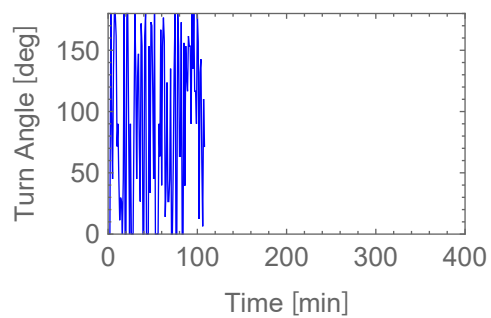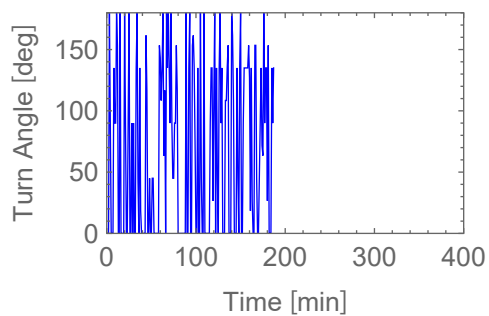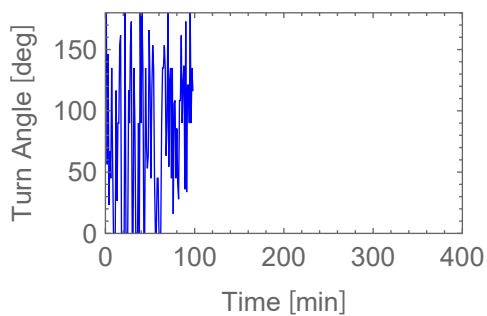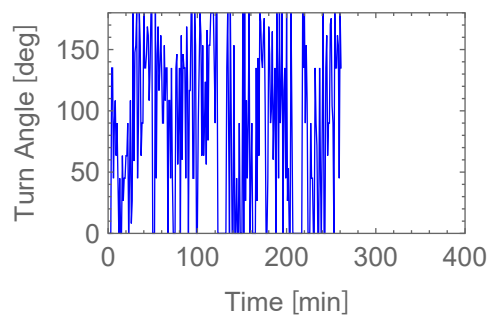

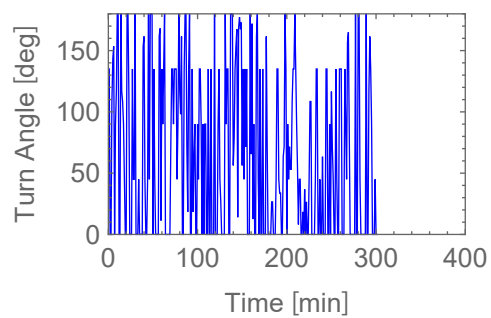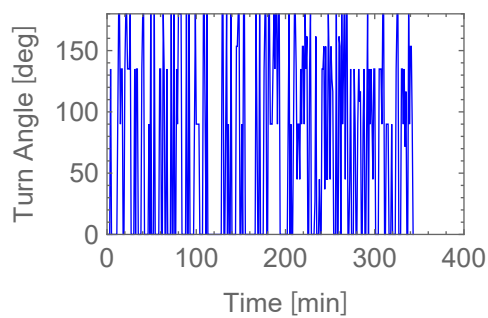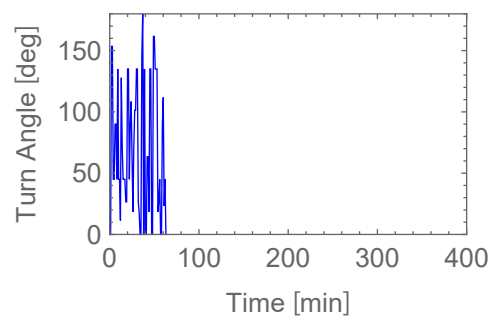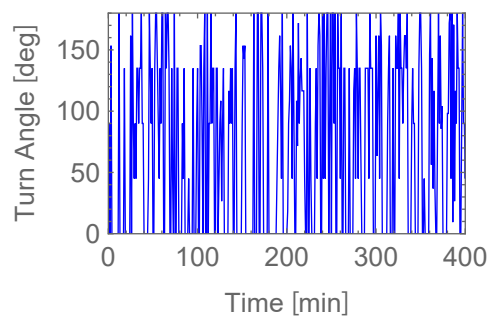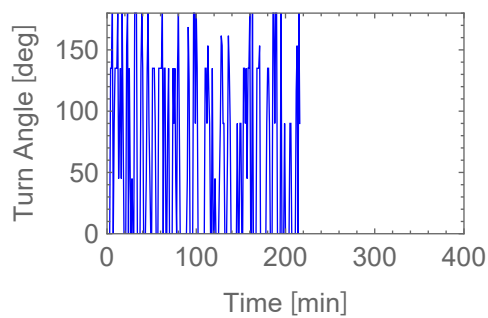

Supplement: S5 Fig — The data were obtained from the same trajectories used in the discrimination analysis. (PDF) [file pone.0320287.s005.pdf]

S6 Fig

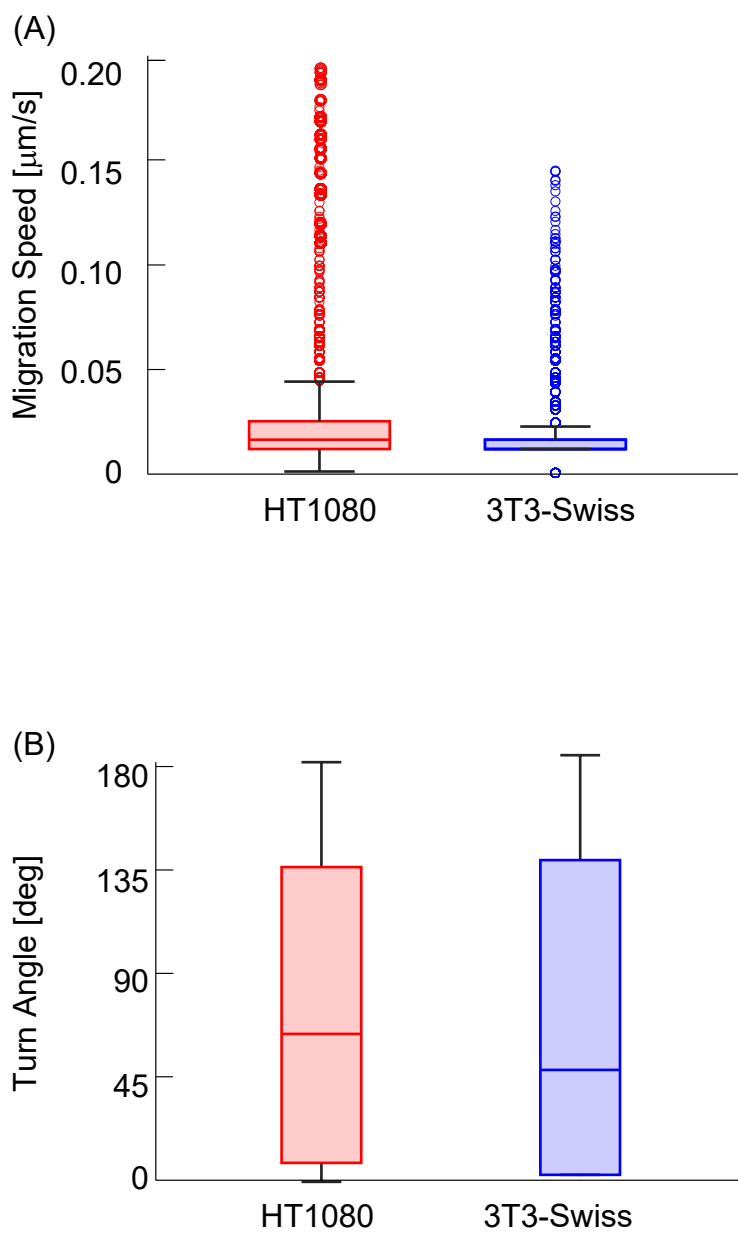

Supplement: S6 Fig — The whiskers represent the lower and upper extremes. Outliers are indicated as single dots. The data of n = 50 HT1080 fibrosarcoma cells (n = 7398 time points) and n = 50 3T3-Swiss fibroblasts (n = 12427 time points) used in the discrimination analysis in Fig. 8 are summarized. (A) Migration speed. Outliers larger than 0.2, specifically n = 1 within 0.24–0.26 in HT1080, and n = 1, 1, and 1 for each within 0.20–0.22, 0.22–0.24, and 0.38–0.40 in 3T3-Swiss are not shown. (B) Turn angle. (PDF) [file pone.0320287.s006.pdf]
