# Supplementary material for: Development of label-free cell tracking for discrimination of the heterogeneous mesenchymal migration
Source: PLoS One. 2025 Mar 31;20(3):e0320287. doi: 10.1371/journal.pone.0320287 (PMC11957292; doi:10.1371/journal.pone.0320287)
Supplement: S1 Table — The parameters were calculated from the cell-tracking data and used in both linear discriminant analysis (LDA) and quadratic discriminant analysis (QDA) for cell-type discrimination. (DOCX) [file pone.0320287.s007.docx]

**Table S1. Cell motility parameters.**

| Parameters |
| --- |
| Migration speed [μm/min] |
| \| Frequency of turns below 90° \| \| --- \| \| Frequency of turns below 60° \| \| Frequency of turns below 30° \| \| Migration distance between turns below 90° [μm] \| \| Migration distance between turns below 60° [μm] \| \| Migration distance between turns below 30° [μm] \| \| Sum of turn angles [deg] \| \| Quiescent time [min] \| \| Total migration length [μm] \| |
