# Supplementary material for: Development of label-free cell tracking for discrimination of the heterogeneous mesenchymal migration
Source: PLoS One. 2025 Mar 31;20(3):e0320287. doi: 10.1371/journal.pone.0320287 (PMC11957292; doi:10.1371/journal.pone.0320287)
Supplement: Attachment — Submitted filename: Rebuttal-Letter-r1.pdf [file pone.0320287.s011.pdf]

PLOS ONE

Ref: PONE-D-24-28597

Title: Development of label-free cell tracking for discrimination of the heterogeneous mesenchymal migration

Response to Editorial Office:

1. Please ensure that your manuscript meets PLOS ONE's style requirements, including those for file naming. The PLOS ONE style templates can be found at https://journals.plos.org/plosone/s/file?id=wjVg/PLOSOne_formatting_sample_main_body.pdf and https://journals.plos.org/plosone/s/file?id=ba62/PLOSOne_formatting_sample_title_authors_affaffiliati.pdf

Response:
We have ensured our manuscript meets PLOS ONE's style requirements.

2. Thank you for stating the following in the Acknowledgments Section of your manuscript:
"This work was supported by Tokyo Metropolitan Government Advanced Research Grant Number R2-2, and TMU strategic research fund for social engagement."
Please note that funding information should not appear in the Acknowledgments section or other areas of your manuscript. We will only publish funding information present in the Funding Statement section of the online submission form. Please remove any funding-related text from the manuscript.

Response:
According to the Editor’s instruction, we have deleted funding information from our manuscript.

3. We are unable to open your Supporting Information files:

S1_Fig.eps
S2_Fig.eps

Response:
We have revised and re-uploaded the files.

Response to Reviewer #1

Reviewer #1: In this paper, the authors developed an automated label-free cell tracking system and compared the motility parameters of cancer and non-cancer cells. They showed that the two cell types can be discriminated by parameters related to turning and argued the usefulness of their high throughput methodology. Figures 5 and 6 summarize multiple motility parameters extracted from the individual tracking data, and Figures 7 and 8 highlight parameters to discriminate cancer and non-cancer cells. Though limited to the two cell types, the methods may contribute to the basic research field. The authors have appeared to validate the method regarding accuracy, sensitivity, and others; however, some concerns remain as listed below.

Response:
We are grateful to Reviewer #1 for the critical comments and useful suggestions that have helped us improve our paper considerably. As indicated in the following responses, we have taken all these comments and suggestions into account in the revised version of our paper. In the revised manuscript, all amendments except for minor corrections in English grammar and stylistic have been indicated in blue. The corrections were made by a native English proofreader in response to the suggestion from one of the reviewers. These corrections did not affect the conclusions.

1.1 The problems of ref.17 is described in the introduction, but the reference also developed label-free tracking applicable for phase-contrast images. Besides this, as perhaps the authors have known, various automated, label-free cell tracking methods for mammalian cells (e.g., DOI:10.1016/j.isci.2022.104678, 10.1109/ICMLA55696.2022.00057, 10.1038/s41598-023-50227-9) and microorganisms (e.g., DOI: 10.1038/s41598-022-27158-y) have been published. The authors should cite some related papers and argue the advantages of the method developed in this study more carefully.

Response 1.1
We wish to express our deep appreciation to the Reviewer for the insightful comment. As the Reviewer pointed out and we also knew, there have been various automated, label-free cell tracking methods. Upon careful examination of the advantages of the method developed in this study compared with related papers, together with the revisions according to other comments from the Reviewer, we specified the advantage of our method as characterization and discrimination of the different mesenchymal modes of movement exhibited by cancer and non-cancer cells on a polystyrene dish. The argument about the advantages of the methods has been moved to the Discussion section in the revised version. In the Introduction, related papers providing background to understand the discussion on these advantages have been cited. The amendments to the

Introduction and the Discussion are as follows.

Introduction:

- The papers reporting label-free phenotyping cells during lineage specification ([9] Kimmel et al., IEEE/ACM Trans. Comput. Biol. Bioinform., 2021; [10] Buggenthin, Nat. Methods, 2017) have been cited to explain that cell motility serves as a good indicator to characterize the cellular state (lines 51–54 on page 4).
- The papers reporting tracking of cells in phase contrast micrographs ([26] Han et al., IEEE ICMLA, 2022; [27] Holme et al., Sci. Rep., 2023; [28] Gu et al., iScience, 2022; [29] Tsai et al., SoftwareX, 2019; [30] Al-Zaben et al., Sci. Rep., 2019, ref 17 in the original version) have been cited (lines 76–84, on page 5) with the description of the strengths and limitations of microscopy for label-free and fluorescently labeled cells. Here, the problem in ref. 30 (Al-Zaben et al., Sci. Rep. 2019, ref. 17 in the original version) pointed out by the Reviewer has been solved.

The argument on the advantages of our method in the Discussion:

The distinguishing point of this study is the successful characterization of different types of mesenchymal modes, specifically a malignant invasive cancer-type and non-malignant fibroblast-type migration, of cells cultured on plastic tissue culture vessel surfaces (lines 399–403 on page 24). We have also provided a basis for discrimination, i.e., the reason why the "sum of turn angles" was, in combination with the other motility parameters of the "frequency of turns below the threshold (30°)," a key parameter to highlight the difference in the migration characteristics of HT1080 fibrosarcoma cells and 3T3-Swiss fibroblasts (lines 451–472 on pages 27–28 in the 5th paragraph in the revised Discussion). The discrimination between two types of mesenchymal mode migration with motile parameters extracted from a label-free image and its basis in relation to the fine-grained difference in the scheme of propulsive force generation is, as far as we know, a first.

In the 8th paragraph of the Discussion (lines 503–521 on pages 29–30), the proposed method and state-of-the-art methods have been evaluated in the context of the requirements of segmentation and tracking to identify the fine-grained characteristics in the cancer type and the non-cancer fibroblast-type mesenchymal migration on flat tissue culture polystyrene, which is the greatest strength of our method. In the paragraph, another review article ([25] Maddalena et al., Algorithms, 2022) is cited in addition to the review articles ([23] Vicar et al., BMC Bioinformatics, 2019; [24] Maska et al, Nat. Methods, 2023) in the original version. Moreover, as specific methods for comparison, CellTraxx (for nucleus, [27] Holme et al. Sci. Rep., 2023), a UNet-based method (for nucleus, [28] Gu et al., iScience, 2022), Bright2Nuc (for nucleus, [41] Atwell et al., Cell Rep. Methods, 2023), TrackMate ([42] Tinevez et al., Methods, 2017; [43] Ershov et al., Nat. Methods, 2022), CellPose ([44] Pachitariu et al., Nat. Methods, 2022), and Usiigaci ([29] Tsai et al., SoftwareX, 2019) have been presented.

1.2 "high throughput" is likely one of the merits of the present method. However, 20 or less cells were measured in Fig 5; measured cell numbers in Fig 6 are unclear. On the other hand, Fig 8 is a summary of 50 cells. The authors should solve the inconsistency of the sample size or carefully explain why they submitted the data from such a small sample size because the gap in the sample size between experiments could make statistical data interpretation difficult.

Response 1.2:

We apologize for the confusion due to the inadequate data presentation. The inconsistency of the sample size in the original version was because the results in Figs. 5 and 6 were selected based on time-course data longer than 60 min, even though the migration trajectory data applied to the discriminant analysis included trajectories less than 60 min. To solve the inconsistency, in the revised version, Fig. 5 (C, D, G, H) and Fig. 6 are replaced with the results for the statistical analysis for all the trajectories (n = 50 cells each for HT1080 and 3T3-Swiss) applied to the discriminant analysis. Additionally, the numbers of cells (n = 50 cells each for HT1080 and 3T3-Swiss) has been added in the figure captions (Fig. 5: lines 296–298 on pages 17–18; Fig. 6: lines 323–325 on page 19).

1.3 Figs 5 and 6 show faster speed of cancer cells than non-cancer cells. The high speed in cancer cells would be due to the spike-like speed increment seen in Fig 5c. Indeed, the transient high speed is distinguished from non-cancer cells, but the cancer cells seem to move at a similar speed as non-cancer cells most of the observation time (Figs C and D show the same peak speed). Isn't it reasonable that the transition between the two-speed modes (major low speed and minor high speed) is the speed property unique to the cancer cell? Please explain (i) why the authors focused on the mean speed and (ii) discuss the possible cause of the high-speed locomotion.

Response 1.3

We appreciate the Reviewer's comment on this point. We agree that the transition between the two-speed modes (major low speed and minor high speed) is the speed property unique to the cancer cell. We had the same idea as the Reviewer during the preparation of the original version. The answers to the specific questions (i, ii) regarding this matter are as follows.

(i) Why do the authors focus on the mean speed?

Based on the idea that the transition between the two-speed modes of migration characterizes cancer cell migration, we created the histograms in Fig. 5C and D in the original version, expecting a bimodal distribution for cancer cells and a monomodal distribution for non-cancer cells. The result revealed that HT1080 migration

on the cell population level was statistically characterized as heterogeneous “migration speed” with a broader unimodal distribution compared with the 3T3-Swiss population rather than a biphasic distribution.

We also tried to detect the two-speed mode with a parameter, “Quiescent Time,” which is the time duration maintaining a speed lower than a threshold (average speed) (Fig. 6E). However, the difference between cancer- and non-cancer cells was not significant, both in the original (the data longer than 300 min) and revised (the n = 50 data applied to the discrimination analysis) versions.

Taken together, as the Reviewer suggested, time-course data for each single HT1080 cell clearly showed that the two-speed mode is one of the features of a cancer cell at the single-cell level. However, at the cell population level, the statistical data demonstrated that mean speed was a parameter that characterizes cancer and non-cancer cells. Accordingly, in the revised version, the 2nd and 3rd paragraphs of “Quantitative characterization of motilities for HT1080 fibrosarcoma cells and 3T3-Swiss fibroblasts” have been amended to clearly show why we focused on the mean speed, which have been added as follows.

The 2nd paragraph:

- The description of the two-speed mode of single cell migration of HT1080 has been added as “HT1080 migration displayed two-speed modes, consisting of transient high speed and slow speed for the rest of the time, whereas 3T3-Swiss migration speed was temporally homogeneous (Fig 5A and 5B; S2 and S3 Figs (lines 265–267 on page 16).”
- The description of the statistical characterization of HT1080 cell migration at the cell population level has been added as “The unimodal distribution, as opposed to a bimodal one, indicates that the high- and low-speed values in the HT1080 two-speed mode are not fixed for the entire population but vary on a cell-to-cell basis within the population (lines 270–273 on page 16).”

The 4th paragraph:

- A description of the intention behind introducing the parameter “quiescent time” has been supplemented as “The ‘quiescent time’ refers to the duration during which a cell maintains a speed lower than a defined threshold (average speed) and was proposed as a potential parameter to differentiate the two-speed mode of HT1080 fibrosarcoma cells from the low-speed maintenance of 3T3-Swiss fibroblasts. However, contrary to expectations, the difference between these two behaviors was not reflected in the average quiescent time values (lines 310–314 on page 18).”

(ii) the possible cause of the high-speed locomotion

From a data analysis viewpoint, the possible cause of the averaged higher value in speed for the HT1080 cell population is that averaging the transient higher values and the low values almost the same as the 3T3-Swiss, yields a higher speed in HT1080 on average than in 3T3-Swiss. From a cell biological viewpoint, a possible cause is described in the 6th paragraph of the Discussion: “The fast-slow migration modes might be driven by

similar mechanisms to those reported in fish keratocyte cells, where migration switches between fast and slow modes due to variations in intracellular diffusion rates and structural changes in the lamellipodium, transitioning between thin and thick morphologies [35 (Jiang et al. Nat. Commun., 2023)] (lines 475–478 on page 28)."

Response to Reviewer #2

Reviewer #2: The authors proposed a method for automatic cell tracking in phase-contrast microscopy images. In detail, they proposed a segmentation process using imaging tools of MATLAB and identified quantitative measures to characterize the migration trajectories. Several tests were performed on fibrosarcoma cells as a cancer mass as well as on normal cell models to validate the proposed method.

Response:
We are grateful to Reviewer #2 for the critical comments and useful suggestions that have helped us improve our paper considerably. As indicated in the following responses, we have extensively revised the manuscript by considering all these comments and suggestions. In the revised manuscript, all amendments except for minor corrections in English grammar and stylistic have been indicated in blue. The corrections were made by a native English proofreader in response to the suggestion from one of the reviewers. These corrections did not affect the conclusions. We would be glad to respond to any further questions and comments that the Reviewer may have.

2.1 In detail, the segmentation process performed with MATLAB does not consider the features of microscopy phase-contrast images (halo, low contrast, and several other imaging artifacts).

Response 2.1
We agree that we should consider the features of microscopy phase-contrast images (halo, low contrast, and several other imaging artifacts). Accordingly, first, in the 3rd paragraph of the Introduction in the revised version, we presented the features usable to birefringent specimen containers (lines 74–76 on page 5), halo artifact for the thick specimen (lines 76–77 on page 5). Then, based on these features, we described the case-by-case points of consideration in the use of tracking tools for such phase-contrast images (lines 77–84 on page 5). The issue regarding the low contrast for the thin specimen has been addressed in the 4th paragraph in the Introduction (lines 89–96 on page 5). We would like to note in particular that the case-by-case points have been presented to highlight points related to the novelty of this study, which is the characterization of the different mesenchymal modes of movement exhibited by cancer cells and non-cancer cells on a polystyrene dish.

2.2 Segmentation and cellular tracking are the pillars of every microscopy image analysis procedure, and the literature on phase-contrast imaging is rich. However, the manuscript does not compare any state-of-the-art methods expecially in the artificial intelligence field. On the other hand, the proposed method has not addressed the main issues in phase-contrast images. For example, the cells have low contrast concerning the

background, complex and different structures varying in time, and touch or overlap. A recent survey can be found here: https://doi.org/10.3390/a15090313

Response 2.2

We agree that we should have compared the proposed method with the state-of-the-art methods, including the issues in phase-contrast images. Accordingly, we have revised the manuscript to include these points. The proposed method and state-of-the-art methods have been evaluated in the context of the requirements of segmentation and tracking to identify the fine-grained characteristics in the cancer type and non-cancer fibroblast-type mesenchymal migration on flat tissue culture polystyrene, which is the most significant aspect of our method. The points of the revision are as follows.

First, a description of the novelty of the research—the discrimination of two types of mesenchymal mode migration with motile parameters—has been added in the first paragraph of the Discussion (lines 399–403 on page 24) with the background (the 2nd paragraph of the Introduction, lines 46–60 on pages 3–4). Additionally, the requirements of segmentation and tracking for the mesenchymal mode discrimination identified throughout the process from the image acquisition to the discrimination have been discussed in the 7th paragraph of the Discussion (lines 484–502 on pages 28–29). Finally, the potential use of the state-of-the-art methods has been discussed in the 8th paragraph of the Discussion (lines 503–521 on pages 29–30), in comparison with the proposed method.

Regarding the issues of low contrast in phase contrast micrographs, complex and different structures varying in time, and touch or overlap we have addressed in the Introduction and Discussion in the revised version:

- Low contrast in phase contrast micrographs: This issue has been addressed as "In cases involving cells with a spreading shape on two-dimensional adhesive surfaces, such as conventional tissue culture polystyrene, the halo effect is minimal. This facilitates well-resolved cell contours at a subcellular scale, especially when combined with image analysis methods capable of detecting the low contrast associated with small phase shifts [29 (Tsai et al, Software X, 2019), 30 (Al-Zaben et al., Sci. Rep., 2019)] (lines 81–84 on page 5)." Accurately capturing low contrast thin lamellipodia while remaining insensitive to subcellular structures such as the organelles is a requirement for the identification of fine-grained characteristics in the mesenchymal migration modes, as discussed in the 7th paragraph of the Discussion (lines 496–502 on page 29).
- Complex and different structures varying in time: Detection of "complete cell tracks at 1-min intervals for over 1 h, corresponding period from one peak to the next in two-speed mode migration of cancerous HT1080 fibrosarcoma (lines 490–491 on page 29)" is a requirement for distinguishing between the mesenchymal migration modes of cancerous fibrosarcoma and non-cancerous fibroblast cell types on flat tissue culture polystyrene. The significance of avoiding fragmentation errors has been addressed along

with the importance of the label-free image acquisition method in the 7th paragraph of the Discussion (lines 491–495 on page 29).

- Touch or overlap: Cell–cell contact is an important issue that supports the basis of characterization and discrimination of heterogeneous mesenchymal mode migrations rather than just for a segmentation method. We feel a deep interest in the influence of cell–cell contact, but it is beyond the scope of this study, which focuses on the discrimination of two types of mesenchymal mode migration with motile parameters extracted from a label-free image based on the fine-grained difference in the scheme of propulsive force generation. We have addressed the influence of cell-cell contact on discrimination accuracy as a topic for further research in the 8th paragraph of the Discussion section of the revised manuscript (lines 525–529 on page 31).

2.3 The quantitative analysis to characterize the phenotypes should be applied to more cell lines for validation.

Response 2.3

We appreciate the Reviewer's interest in the extensibility of the quantitative analysis to characterize the phenotypes. But first of all, the distinguishing point of this study is the successful characterization of different types of mesenchymal mode, specifically a malignant invasive cancer-type and non-malignant mesenchymal-type migrations of cells cultured on plastic tissue culture vessel surfaces (lines 399–403 on page 24). Additionally, we have provided a basis for discrimination, i.e., the reason why the “sum of turn angles” was, by combining it with the other motility parameters of the “frequency of turns below the threshold (30°),” a key parameter to highlight the difference in the migration characteristics of HT1080 fibrosarcoma cells and 3T3-Swiss fibroblasts in the 5th paragraph of the revised Discussion (lines451–472 on pages 27–28). The discrimination of two types of mesenchymal mode migration with motile parameters extracted from a label-free image with its basis in relation to the fine-grained difference in the scheme of propulsive force generation is, as far as we know, a first. We believe that the clarification of the strategy and the basis to identify the difference expands the possibilities for basic research and medical engineering applications of label-free cell segmentation and tracking, and the finding regarding the basis itself has significant implications for the cell biological field. We do agree that additional examination for more cell lines is valuable but would like to keep the focus intact. Thus, the validation of expandability has been included in the revised manuscript as a consideration for future study (lines 541–543 on page 32).

2.4 In my opinion, the manuscriopt appears in an early stage and requires significant improvements to be ready for publication.

Response 2.4

We regret our insufficient consideration of the cell discrimination and description of the novelty of the study in the original version. In response to the Reviewer’s comments, we deeply re-examined the basis of the discrimination. Consequently, we noticed that the novelty of this study is not just two types of cell discrimination methods with the proposed segmentation and tracking. Instead, as also described in the above response 2.3, a major point is the discrimination of two types of mesenchymal mode migration with motile parameters extracted from a label-free image with its basis in relation to the fine-grained difference in the scheme of propulsive force generation. We believe that the clarification of the strategy and the basis to identify the difference in two types of mesenchymal migration modes—malignant invasive cancer-type and non-malignant fibroblast-type—expand the possibilities for basic research and medical engineering applications of label-free cell segmentation and tracking, and the finding regarding the basis itself has important implications for the cell biological field. We have extensively revised the manuscript. The points of the revision are as follows.

Title: It has been revised to “Development of label-free cell tracking for discrimination of the heterogeneous mesenchymal migration,” which concisely represents the novelty of this study, from “Development of label-free cell tracking for cell discriminant analysis based on cell motility.”

Abstract: We have added a description regarding the discrimination of two types of mesenchymal mode migration (lines 19–21 on page 2) with motile parameters extracted from phase-contrast label-free images with its basis in relation to the fine-grained difference in the scheme of propulsive force generation (lines 23–29 on page 2).

Introduction:

- The description regarding heterogeneity in cell motile behavior and the previous research on discrimination has been added in the 2nd paragraph (lines 46–60 on pages 3–4, please refer also to response 2.2).
- The description of consideration at the starting point in the experimental design based on the cell segmentation and tracking approach has been added to the 3rd paragraph (lines 61–62 on page 4; lines 67–84 on pages 4–5). In the paragraph, case-by-case points of consideration in the use of segmentation and tracking tools are described (please refer also to response 2.1).
- The description of the presumed requirements to distinguish heterogeneity of mesenchymal mode migrations, which would be crucial for the readers to understand the novelty and importance of our methodology, has been added to the last paragraph (lines 85–96 on pages 5–6).
- We have added a concrete statement regarding the novelty of our research as “Additionally, based on the results of the discriminant analysis, we identified the requirements for segmentation and tracking methods necessary to effectively process and extract the characteristics of mesenchymal mode migration. This

includes capturing detailed differences to distinctly differentiate between the malignant HT1080 fibrosarcoma cells and the non-malignant 3T3-Swiss fibroblasts (lines 100–104 on page 6)" at the end of the last paragraph.

Discussion:

- We have additionally included a statement about the novelty and importance of this study, specifically, the distinguishing point of this study, successful characterization and discrimination of different types of mesenchymal modes in the first paragraph (lines 399–403 on page 24).
- Two paragraphs describing the basis of the characterization and discrimination (lines 442–472 on pages 26–28) and another presenting the requirements for success (lines 484–502 on pages 28–29) have been supplemented.
- Our method has been compared with the state-of-the art segmentation and tracking methods with the objective of the characterization and discrimination of different types of mesenchymal modes (lines 503–521 on pages 29–30).
- We also have addressed limitations and future scope, regarding the need for validation of the effect of cell-cell contact (lines 525–529 on page 31), expandability to various types of cells (lines 541–543 on page 32) for future study.

Response to Reviewer #3

Reviewer #3: The authors present an automatic tracking method for phase-contrast images of fibroblast cells, which works label-free and is capable to discriminate different cell lines according to motility Parameters, which are used as input for linear and quadratic classification methods. The method is interesting, while the findings of the authors should be clarified in more detail.

Response:
We are grateful to Reviewer #3 for the critical comments and useful suggestions that have helped us improve our paper considerably. As indicated in the following responses, we have taken all these comments and suggestions into account in the revised version of our paper. In the revised manuscript, all amendments except for minor corrections in English grammar and stylistic have been indicated in blue. The corrections were made by a native English proofreader in response to the suggestion from one of the reviewers. These corrections did not affect the conclusions.

3.1 In line 66 on page 11 (Introduction). The authors Mention 3t3-Swiss fibroblasts. The name of the cell line was differently written in the abstract of the presented work (line 22 of page 9).

Response 3.1
Thank you for pointing this out. We have amended the name of the cell line in the Abstract and unified it to "3T3-Swiss."

3.2 In line 78 on page 11. the letter 'phi' for the Diameter of a polystyrene dish is confusing?

Response 3.2
We have rephrased the words as "in a polystyrene dish (35 mm, VIOLAMO)" in the revised version (line 113 on page 7).

3.3 Authors mention an automatic tracking tool, but use a manually adjusted threshold Adjustment (line 101 on page 12)?

Response 3.3
Thank you for your comment. The threshold of the first of the images was adjusted manually. Once adjusted, the same threshold can be used for the same imaging setup: a CMOS camera (ORCA-Flash 4.0, Hamamatsu Photonics), a 10× 0.30 NA objective lens (UPLFLN10X2, Olympus), and an inverted microscope (IX83,

Olympus), regardless of HT1080 and 3T3-Swiss. Accordingly, the explanation was supplemented as follows: "Once the threshold was manually adjusted for an image, the same value was applied regardless of whether it was HT1080 fibrosarcoma cells or non-cancer 3T3-Swiss fibroblasts for the same image acquisition setup (lines 139–141 on page 8)."

3.4 Fig 1B, It is not clear to the reader how the image processing from 'Fill' to 'Erode' reduce in dimension? In Addition. The 'original' and the 'convex hull' images are very different, why?

Response 3.4

We should further explain the image analysis step in the original version. In the revised version, we have supplemented the explanation in Materials and Methods as follows:

Lines 141–145, on page 9: "Next, the binarized images were dilated, and holes were filled. The dilation was performed using an octagonal structuring element with a radius of 3 pixels (1.95 μm) from the origin to the sides of the octagon, measured along the horizontal and vertical axes. In addition, erosion was performed twice using a diamond structuring element with 5 pixels (3.25 μm) from the origin of the structural element to the point of the rhombus."

We apologize for the mistake regarding the difference between the "original" and the "convex hull" images. We have corrected the "convex hull" image in Fig. 1B.

3.5 In line 176 on page 16 (Results). The authors mention that the routine stops when a cell Encounter another one. How this event is detected automatically? by the area of the cell?

Response 3.5

If a cell encounters another cell, either no centroid or more than one cell centroid is given, and the routine stops. The details have been supplemented as follows: "In these cases, where either no centroid or more than one cell centroid was detected, cell tracking was stopped automatically at that point and the tracking data before contact were used for analysis (lines 234–236 on page 14)."

3.6 What is the average size and area of the investigated cells?

Response 3.6

The average equivalent circle diameters are 47.2 ± 9.7 μm (mean ± SD, n = 50, HT1080 fibrosarcoma) and 52.2 ± 7.9 μm (mean ± SD, n = 50, 3T3-Swiss fibroblast). The average spreading areas are 1865.9 ± 871.8 μm^2 (mean ± SD, n = 50, HT1080 fibrosarcoma) and 2251.8 ± 618.7 μm^2 (mean ± SD, n = 50, 3T3-Swiss

fibroblast). The explanation was supplemented (lines 222–225 on page 13).

3.7 Authors mention 5 typical trajectories. These ones have been chosen with which criteria? (line 196 on page 17)

Response 3.7

We agree that this point requires clarification. The criteria were ambiguous in the original version, and the 5 typical trajectories were re-selected based on the following criteria in the revised version:

HT1080 fibrosarcoma: "Trajectories with two-speed modes and an average migration speed of 0.02 μm/s or high are shown as typical (lines 256–257 on page 15)."

3T3-Swiss fibroblast: "Trajectories with an average migration speed of 0.015 μm/s or low are shown as those typical (lines 259–260 on page 15)."

3.8 In line 205 on page 18 the authors mention a lower Migration speed of 3t3-Swiss cells. This value is the median value? Which parameter was considerate to see if these 2 datasets are significantly different?

Response 3.8

We apologize that this part was not clear in the original version. The description "a lower migration speed of 3T3-Swiss cells" did not refer to a quantitative value such as the median value. The description referred to a qualitative observation about the pattern in the time course of migration speed in Figs. 5A (HT1080) and 5B (3T3-Swiss). More precisely, HT1080 cells had two-speed modes: transient high speed, and a similar speed as non-cancer 3T3-Swiss fibroblasts for the rest of the time. To specify the feature of the pattern in the time course of the migration speed, we have amended the description (lines 264–267 on page 16) in the Results section, "Quantitative characterization of motilities for HT1080 fibrosarcoma cells and 3T3-Swiss fibroblasts."

3.9 In Fig. 5c & d a box chart plot would maybe better represent the shown data.

Response 3.9

We thank the Reviewer for this comment. We have created box plot chart plots and supplemented them in S6 Fig. Although we consider that the box plot chart is effective in grasping the distribution of outliers, the histograms in the original version are retained in the revised version for the following reasons:

Regarding the histogram in the original version, we intended to explore the parameter(s) to quantitatively describe the two-speed modes, a feature of HT1080 fibrosarcoma migration. Originally, we expected HT1080 fibrosarcoma cells having two-speed modes could characterize a bimodal distribution, in contrast with a

monomodal distribution of non-cancer 3T3-Swiss fibroblasts. However, the histogram for HT1080 fibrosarcoma was not a bimodal distribution, but rather a broader distribution in the cancer cells than in the non-cancer cells. Despite this result, the broader distribution is still important to quantitatively characterize the two-speed mode of HT1080 fibrosarcoma at the cell population level. Regarding the angle change, the histogram is important to discuss the precision of the measurement: "The histograms reflect the effect of discretizing centroid displacement, where cell centroid movements per unit time are limited to one of the adjacent pixels, resulting in peaks at approximate angles of 0°, 45°, 90°, 135°, and 180° (lines 287–289 on page 17, English has been corrected according to another Reviewer's comment)."

3.10 In line 220 on page 18 the authors mention the pixel resolution. What is the pixel resolution of this experiment?

Response 3.10

The pixel resolution, 0.65 μm/pixel, was added in line 286 on page 17.

3.11 Why the authors use only 2 points to calculate the turning angle? More datapoints could give a more consistent turning angle? The choice of 2 data-points for the angle calculation is due to the characteristic time of a turning angle? What is the error of such a measurement?

Response 3.11

We thank the Reviewer for this comment. We selected the time interval of 1 min to capture the changes in centroids associated with morphological changes at the cellular level in addition to cell displacement. Whether the effect of the morphological change involved in the turn angle is an error source or potentially meaningful information will vary depending on the purpose, thus we cannot uniquely address the error of the measurement with a 1-min interval of 2 data points. Within the scope of this cancer cell discrimination, the time scale at a minute level is effective. To make this point clearer, we have amended the Discussion by adding the discussion on the time scale of the motility parameters (lines 423–429 on page 25). Although we think it would be valuable to further examine the relationship between parameters acquired over a wide range of time scales and cell identification accuracy, it falls beyond this study. We would like to report it in a later paper.

3.12 In line 234 on page 19. the authors mention a 1.5 times higher migration speed? How it looks for the median value?

Response 3.12

We apologize for the lack of explanation on the values in Fig. 6. The values are mean + SD, thus the description is based on the mean value. The statistical significance between the HT1080 fibrosarcoma and 3T3-Swiss fibroblast has also been evaluated using the Welch t-test in the revised version. To clarify, we have added the following text to the Results:

Lines 301–303 on page 18: "The mean migration speed (Fig 6A) of HT1080 cells was approximately 1.5 times higher than that of 3T3-Swiss fibroblasts (also 1.5 times in the median value, S6 Fig.)."

Caption of Fig. 6, lines 323–325 on page 19: "Data are mean + SD for n= 50 HT1080 cells and n = 50 3T3-Swiss fibroblasts used for the discrimination analysis in Fig 8. *P*-values were calculated using the Welch t-test: $*P < 0.05$, $**P < 0.01$, $***P < 0.001$."

3.13 Authors mention no differences for 'sum of turns', but later on in line 256 on page 20 mention the most remarkable difference for 'sum of turns'. The 'sum of turn angle' has a quite big variance. I do not understand the physical meaning of this parameter (sum of turn angle). The authors could provide more information.

Response 3.13

We wish to express our deep appreciation to the Reviewer for the insightful comment on this point to improve the manuscript. As the Reviewer pointed out, the "sum of turn angle" has quite a big variance within each population of the HT1080 fibrosarcoma cells and 3T3-Swiss fibroblasts. However, noticeably, the correlation analysis demonstrated that the "sum of turn angle" was, in combination with the other motility parameters of the "frequency of turns below the threshold (30°, 60°, 90°)" and the "migration speed," useful to highlight the difference in the migration characteristics of HT1080 fibrosarcoma cells and 3T3-Swiss fibroblasts. We believe that the difference in the way of correlation is based on the process to attain effective cell body propulsion. Accordingly, we have amended the Results and the Discussion as follows:

Results: Text (lines 333–336 on page 10) has been amended to clearly show the point regarding the "sum of turn angles" as "Although the 'sum of turn angles' alone exhibited large variance and no significant difference between HT1080 fibrosarcoma cells and non-cancer 3T3-Swiss fibroblasts (Fig 6D), its correlation with 'migration speed' and 'frequency of turns below thresholds' differed between these cell types."

Discussion: We have extensively revised the third paragraph discussing cell discrimination with the correlation coefficients to be more specific. In the revised version, the paragraph has been divided into three paragraphs, and the physical meaning of the "sum of turn angles" has been added in the 5th paragraph as follows:

- The physical meaning was supplemented as "We interpret the 'sum of turn angles' as the cumulative degree of polarity changes caused by cell deformation (lines 454–455 on page 27)."
- The meaning of the correlation of the "sum of turn angles" with "migration speed" and "the frequency of

turn under a threshold (30°, 60°, 90°)" was supplemented from the viewpoint of the way to obtain effective cell body displacement in relation to the cell polarity changes: "Together with the negative correlation with "migration speed," the "sum of turn angles" in combination with the "frequency of turns below the threshold (30°, 60°, 90°)" reflects a distinguishing mechanism in HT1080 fibrosarcoma cells. In this mechanism, effective cell body propulsion is attained through cell deformation while preserving polarity. In contrast, in 3T3-Swiss fibroblast migration, the "sum of turn angles" showed a positive correlation with "migration speed," but no correlation with "frequency of turns below the threshold (30°, 60°, 90°)" (Fig 7). This pattern suggests that the relationship between the "sum of turn angles," "frequency of turns below the threshold," and "migration speed" reflects a mechanism by which effective cell propulsion is achieved through cell deformation accompanied by polarity changes, regardless of their magnitude (lines 464–472 on pages 27–28)."

3.14 Why the authors limited their classification to 2 classifiers? A Support Vector Machine classifier would maybe provide a better outcome?

Response 3.14

We thank the Reviewer for this comment. Although we think it would be valuable to provide a better outcome to further examine the effectiveness of support vector machine classifiers, it falls beyond the scope of this study, which aims to assess the possibility of cell motility-based discrimination with the requirements of spatiotemporal resolution for segmentation and tracking. We would like to only discuss the potential use of the support vector machine as a future issue in the Discussion (lines 524–525, on page 31, the 8th paragraph) and report it in a later paper.

Response to Reviewer #4

Reviewer #4: The authors propose a method for tracking cells present in phase contrast images and performed discrimination of cell types based on trajectory characteristics with potential applications in cancer, tissue healing, and other areas.

Response:
We are grateful to Reviewer #4 for the critical comments and useful suggestions that have helped us improve our paper considerably. As indicated in the following responses, we have taken all of these comments and suggestions into account and have extensively revised the manuscript. In the revised manuscript, all amendments, except for minor corrections in English grammar and style, have been indicated in blue. The corrections were made by a native English proofreader in response to the Reviewer's suggestion. These corrections did not affect the conclusions. We would be glad to respond to any further questions and comments that the Reviewer may have.

4.1 The authors state that "Cell phenotyping based on static data **should be** complemented by dynamic data extracted from time-dependent cell characteristics" but this depends on the study and application. Maybe a better assertion is "For some studies it is important that cell phenotyping based on static data ** be ** complemented by dynamic data extracted from time-dependent cell characteristics". Please revise.

Response 4.1
The Reviewer's comment is correct. Accordingly, we have revised the corresponding sentence (line 17, page 2 in the Abstract; line 45, page 3 in the Introduction).

4.2 "Additionally, if it is a deep-learning strategy, huge training data is indispensable". This is a misleading assumption. Pre-trained models have come a long way and they can be effectively fine tuned using a handful of new annotations (new ground truth) to render excellent segmentation solutions on new datasets at hand. It might not take more than a day or so to annotate a small set of new images with hundreds of cells to obtain a successful fine tuned model for your data. Readily available tools like Cellpose, StarDist, Omnipose are popular and proven ones adopted by many. They have been used in many image categories including phase contrast images. The authors need to compare their methods with results from these developments to justify proposing their new segmentation strategy.

Response 4.2
We regret the misleading assumption regarding the training data of the deep learning strategy. Accordingly,

the description has been deleted in the revised version. Additionally, we agree that we need to compare readily available preceding tools with our method. Accordingly, we compared the methods as follows.

- First, we identified the requirements of segmentation and tracking tools to identify the fine-grained characteristics of the cancer type and a non-cancer fibroblast-type mesenchymal migration on flat tissue culture polystyrene, which is the greatest strength of our method (lines 484–502 on pages 28–29 in the 7th paragraph of Discussion).
- Then, the effectiveness of the preceding tools has been discussed from the viewpoint of whether the tools fulfill the requirements (lines 503–529 on pages 29–31 in the 8th paragraph in Discussion).

4.3 Deep learning based methods have dominated in cell segmentation tasks - see, for example, top ranking segmentation methods in the publication cited by the authors "The Cell Tracking Challenge: 10 years of objective benchmarking", more specifically the Cell Segmentation Benchmark. Such evidence, and others, greatly weakens the development of cell segmentation schemes using classical methods (Sobel filter, morphological dilation and erosion, thresholding), as it is done by the authors. In special, manually adjusting thresholds ("...manually adjusted [threshold] for each cell type") is not favorable and quite frankly not needed as the methods mentioned above eliminate such step. My strong suggestion is for the authors to adopt these other more robust and proven methods to obtain the segmentations they need and eliminate their proposed method, which might not work, for example, for close proximity cells.

Response 4.3

We strongly appreciate the Reviewer's comment on this point.

We agree that segmentation and tracking packages eliminate the manual processes, thus, we added the description in the 8th paragraph comparing our method with others in the Discussion as "Other deep learning-based tracking and segmentation packages, such as Usiigaci [29 (Tsai et al. SoftwareX, 2019)], can be used if properly trained with appropriate datasets. ….. Furthermore, state-of-the-art deep learning-based methods are expected to reduce manual processes, such as threshold adjustment in edge detection in our method (Fig 1) (lines 515‑521 on page 30)."

Regarding the segmentation of proximity cells, we agree that deep learning-based segmentation and tracking packages might work well. However, the inclusion of cell behavior before and after contact into the discriminant analysis possibly obscures the cancer fibrosarcoma cell-specific way of cell body propulsion that is "attained through cell deformation while preserving polarity" (line 467 on page 27), resulting in decreasing discrimination accuracy. We feel a deep interest in the influence of cell–cell contact but it is beyond the scope of this study that focuses on the discrimination of two types of mesenchymal mode migration with motile parameters extracted from a label-free image with its basis in relation to the fine-grained difference in the scheme of propulsive force generation. We have included the description of the influence of cell-cell contact

on discrimination accuracy as a topic for further research in the 8th paragraph of the Discussion section of the revised manuscript (lines 525–529 on page 31).

Regarding the Reviewer's comment #3 as a whole, we regret the insufficient consideration of cell discrimination and poor description of the novelty of this study. In response to the comment, we deeply re-examined the basis of the discrimination. Consequently, we noticed that the novelty of this study is not just development segmentation and tracking. Instead, a major point is the discrimination of two types of mesenchymal mode migration with motile parameters extracted from label-free images with its basis in relation to the fine-grained difference in the scheme of propulsive force generation.

4.4 The literature on image segmentation is very poor and does not reflect the state of the art. There is a single reference to a review article [26] but none that actually presents contemporary cell segmentation methods. The authors must update the literature.

Response 4.4

We agree that we should have presented contemporary cell segmentation methods. Accordingly, we have added a paragraph (lines 503–521 on pages 29–30) in the Discussion to present the contemporary cell segmentation methods in the context of a comparison with the proposed method. In the paragraph, another review article, [25] (Maddalena et al., Algorithms, 2022), is cited in addition to [23] (Vicar et al., BMC Bioinformatics, 2019) and [24] (Maska et al., Nat. Methods, 2023), in the original version. Moreover, as specific methods for comparison, CellTraxx [27] (Holme et al. Sci. Rep., 2023), Usiigaci [29] (Tsai et al., Software, 2019), CellPose [44] (Pachitariu et al., Nat. Methods, 2022), a UNet-based method (for nucleus, [28] Gu et al., iScience, 2022), and Bright2Nuc (for nucleus, [41] Atwell et al., Cell Rep. Methods, 2023) have been presented.

4.5 The literature is also very poor on tracking methods and there is no justification why not adopting other popular methods readily available, e.g. TrackMate, in tools like ImageJ/Fiji. While it might personally be a valuable algorithmic experience, the proposed tracking method does not offer any new novelty to be considered for publication at Plos ONE.

Response 4.5

We regret the insufficient explanation about the novelty of our research. As the Reviewer described, the novelty of this study is not introduced by the proposed segmentation and tracking method alone. Instead, the novelty of our research is the successful discrimination of distinct types of mesenchymal mode, specifically a malignant invasive cancer-type, and well-characterized non-malignant fibroblast-type, migration of cells cultured in plastic tissue culture vessels. We believe it has an impact on the cell biological field that we

achieved the quantitative discrimination of malignant invasive fibrosarcoma and non-malignant fibroblast by characterizing dynamic cell motility and provided the explanation based on the difference in the way of effective propulsive force generation. From the viewpoint of the development of tools, not only was the discrimination successful, but the requirements for the method were also clarified. We believe that the finding greatly expands the possibilities for basic research and medical engineering applications of label-free cell segmentation and tracking, regardless of whether or not our proposed tool is used.

Accordingly, we revised the descriptions regarding the novelty in the Introduction and the Discussion as follows.

- The last paragraph of the Introduction: The description of the heterogeneity of mesenchymal mode migration has been added as "In this study, we aimed to investigate the potential to differentiate between the cancer cell line HT1080 fibrosarcoma and the non-cancer cell line 3T3-Swiss fibroblast. Both cell lines exhibit mesenchymal mode migration when migrating as single cells [5] (Friedl and Wolf, J. Cell Biol., 2010); however, their mesenchymal migration mode migrations are assumed to display heterogeneity due to differences in malignancy, such as the presence or absence of invasiveness (lines 85–89 on pages 5–6)."
- The 7th paragraph of the Discussion: The point for successful discrimination of the heterogeneity has been supplemented as "The key to successfully identifying fine-grained differences in heterogeneous cell motility lies in the spatiotemporal resolution needed to capture both cell body displacements and polarity changes associated with morphological alterations (lines 485–487 on page 28–29)." Then, we described the requirements to identify the cancer fibrosarcoma and a non-cancer fibroblast-type of mesenchymal mode migrations (lines 487–502 on page 29).
- The last paragraph of the Discussion: The description of the applicability has been amended to a more specific one (lines 532–544 on pages 31–32).

4.6 "The tracking was stopped when cell division or contact with other cells occurred". How is this done and computed? It is not clear how this step is achieved. Please elaborate so readers are able to replicate your method.

Response 4.6

In these cases, two centroids or no centroid for the tracking cell of interest were computed and used as the criterion. The description has been added to the Methods (lines 133–136 on page 8).

4.7 Similarly, how does the method decide if segmentation mistakenly generated a single cell, as mentioned in "Moreover, if cell-cell contacts occurred during cell tracking, this method recognized the cells as a single cell

(S1B Fig.). In these cases, cell tracking was stopped at that point and the tracking data before contact were used for analysis"? Please explain.

Response 4.7
When cell segmentation mistakenly generated a single cell due to cell contact, two centroids were computed. The description has been added (lines 234–236 on page 14).

4.8 Typo: "The parameters *are* affected depending on the total tracking time".

Response 4.8
This typo has been corrected in accordance with the Reviewer's comment (line 170 on page 10).

4.9 Please explain the conversion mentioned in "We converted the parameters into those that were tracked for 30 h".

Response 4.9
We agree we should have explained the conversion. The parameters were normalized to values per 30 h by multiplying the hourly values by 30 for statistical and discrimination analysis (lines 170–172 on page 10).

4.10 What do LDA and QDA stand for? Linear and quadratic discriminant analysis? If so, please make it explicit so one can readily understand these acronyms.

Response 4.10
Complete notations for the acronyms LDA and QDA have been provided in their first appearance in accordance with the Reviewer's comment (lines 185–186 on page 11).

4.11 "HT1080 cells was transient increases in the migration speed (Figs. 5A; S2A Fig.)". Please rephrase, it is not understandable. Same for the following, "In both cell types, the peaks at 0, 45, 90, 135, and 180 are existed. This is due to pixel resolution where the cell centroid migrated to one of the adjacent pixels, the turn angles were discrete value of 0, 45, 90, 135, or 180".

Response 4.11
We are sorry for the confusion. We have amended the descriptions as follows.

- Original: “HT1080 cells was transient increases in the migration speed (Figs. 5A; S2A Fig.)”
  Revised: “Fig 5 A and B, respectively, shows typical time courses of migration speed in HT1080

fibrosarcoma cells and 3T3-Swiss fibroblasts. HT1080 migration displayed two-speed modes, consisting of transient high speed and slow speed for the rest of the time, whereas 3T3-Swiss migration speed was temporally homogeneous (Fig 5A and 5B; S2 and S3 Figs.). (Lines 264–267 on page 16).”

- Original: “In both cell types, the peaks at 0, 45, 90, 135, and 180 are existed. This is due to pixel resolution where the cell centroid migrated to one of the adjacent pixels, the turn angles were discrete value of 0, 45, 90, 135, or 180”

  Revised: “In the histograms of turn angles, the probabilities were high within the ranges of 0–20°, 40–50°, 90–100°, 130–140°, and 170–180° for both HT1080 cells and 3T3-Swiss fibroblasts (Fig 5G and 5H). This pattern is attributed to the significant figures used in centroid calculations. Specifically, the analysis was performed based on centroid data calculated in pixels (0.65 μm/pixel). The histograms reflect the effect of discretizing centroid displacement, where cell centroid movements per unit time are limited to one of the adjacent pixels, resulting in peaks at approximate angles of 0°, 45°, 90°, 135°, and 180° (Lines 283–289 on page 17).”

4.12 Typo: "*Calculation* of cell centroids". And many other typos throughout the manuscript. Please revise and correct grammar as well.

Response 4.12

The typo in Fig. 1 has been corrected. According to the Reviewer’s suggestion, the manuscript has been reviewed by a professional English correction service and revised to improve readability.

4.13 The authors state in their conclusion that "Therefore, this method for cell discrimination and motility-based analysis of the cell status is easy to use and inexpensive enabling anyone without professional skills of image analysis, such as algorithm selection and pre- and post-processing, huge training data and special devices. Furthermore, it is highly versatile and can be applied in basic cell biological research and biomedical applications", but there is no evidence of usage in different image categories, different cell morphologies and sizes, and crowded situations where tracking is more challenging.

Response 4.13

We agree that we should have discussed the range of applications of this method with evidence. As the Reviewer indicated, the claim that “it is highly versatile and can be applied in basic cell biological research and biomedical applications” has the risk of overselling and was removed from the conclusion. Instead, the range of the application has been specified as “We evaluated the potential application of this method and demonstrated its usefulness as a novel tool for label-free discrimination of different types of mesenchymal mode migration. The feasibility of this approach was validated through its application to HT1080

fibrosarcoma and 3T3-Swiss fibroblast, both of which are known to exhibit mesenchymal mode migration when migrating as single cells on the surface of plastic tissue culture vessels (lines 399–403 on page 24)" in the first paragraph of Discussion. Furthermore, the limitations in terms of expandability have been discussed as "Expanding the label-free method to characterize cell migration, including the underlying mechanism of propulsive force generation, for a variety of cell types is an important consideration for future research. (lines 541–543 on page 32)" in the 9th paragraph of the Discussion.

4.14 Unfortunately, the authors show a considerable lack of maturity and poor investigation of availability of computing methods to tackle the image processing problem.

Response 4.14
We agree with the Reviewer's comment. To improve the manuscript, we closely re-examined the strengths and limitations of our methods. Consequently, we understood that the distinguishing point of this study was the successful characterization of different types of mesenchymal mode, specifically a malignant invasive cancer-type and non-malignant fibroblast-type, migration of cells cultured on plastic tissue culture vessel surfaces (lines 19–21 on page 2 and lines 399–403 on page 24). Additionally, we have drawn the basis of discrimination in relation to the fine-grained difference in the scheme of propulsive force generation, to the best of our knowledge, for the first time. Accordingly, the manuscript has been extensively revised to focus on this point. Please also see responses 4.5 and 4.15.

4.15 The authors should probably concentrate their contribution on the discriminant analysis beyond the two datasets they present in the manuscript. I am not familiar with the quantitative characterization of cell motilities in different species but hopefully what the authors have achieved in this specific point can be valuable and extended to other types of cells besides HT1080 and 3T3-Swiss.

Response 4.15
We strongly appreciate the Reviewer for the comment. We agree that we should have concentrated our contribution on the discrimination analysis. Accordingly, we deeply re-examined the basis of the discrimination of two types of mesenchymal mode migration with motile parameters extracted from phase-contrast label-free images. Consequently, we clarified that the parameters involve a fine-grained difference in the scheme of propulsive force generation. Thus, in the revised version, a paragraph to describe the basis of the discrimination has been added in the Discussion (5th paragraph, lines 451–472 on pages 27–28). The discrimination of two types of mesenchymal mode migration with motile parameters extracted from label-free images with its basis is, as far as we know, for the first time. We believe that the clarification of the strategy and the basis to identify the difference expands the possibilities for basic research and medical engineering

applications of label-free cell segmentation and tracking, and the finding regarding the basis itself has an important implication for the cell biological field. We do agree that additional examination for more cell lines is valuable but would like to keep the focus intact. Thus, the validation of expandability has been included in the revised manuscript as a consideration for future study (lines 541–543 on page 32).
